# Noble Metal Aerogels: Synthesis and Application as Support-Free Anode Electrocatalysts for Ethanol Electro-Oxidation

**DOI:** 10.3390/gels12050397

**Published:** 2026-05-03

**Authors:** Shaik Gouse Peera, Mohanraj Vinothkannan, Shaik Ashmath, Tae Gwan Lee, Myunghwan Byun, Seung Won Kim

**Affiliations:** 1 Natural Science Research Institute, College of Natural Sciences, Keimyung University, Daegu 42601, Republic of Korea; 2 Centre for Advanced Low Carbon Propulsion Systems (C-ALPS), Centre for E-Mobility and Clean Growth, Coventry University, Coventry CV1 2TL, UK; ae4313@coventry.ac.uk; 3 Department of Environmental Engineering, Keimyung University, Daegu 42601, Republic of Korea; shaikashmath2@gmail.com (S.A.); wateree@kmu.ac.kr (T.G.L.); 4 Department of Advanced Materials Engineering, College of Engineering, Keimyung University, Daegu 42601, Republic of Korea; myunghbyun@kmu.ac.kr

**Keywords:** metal aerogels, support-free catalysts, anode electrocatalyst, ethanol electro-oxidation, direct ethanol fuel cells (DEFCs)

## Abstract

Self-sustained metal aerogels are emerging as advanced porous materials with a 3D network of nanostructures that are exclusively made of metals. Metal aerogels possess a distinctive combination of metallic nanoparticles with excellent electronic conductivity, and the excellent porosity of the aerogels allows the extensive exposure of electrocatalytic active sites, together with remarkable mass transport networks in a single entity, unlocking widespread application potential ranging from energy storage and conversion to environmental remediation. In this review, we systematically examine the potential of metal aerogels as electrocatalysts for ethanol electro-oxidation. Various synthesis routes, structure–property relationships, and their function as anode electrocatalysts have been critically reviewed. Due to their 3D porous metallic nature, noble metal aerogel catalysts were found to exhibit excellent ethanol oxidation currents, anti-poisoning for reaction intermediates, high mass, and specific activities of 5–20 times those of traditional Pd/C catalysts. In conclusion, it is shown that metal aerogel catalysts exhibit enhanced activity for ethanol electro-oxidation currents over traditional Pd/C catalysts. Despite this, several challenges exist in realizing the commercial applications of metal aerogels, which have been clearly and elaborately stated as future perspectives and research directions in the field of metal aerogel electrocatalysis.

## 1. Introduction

Fuel cells are electrochemical devices that convert chemical energy into electricity through electrochemical reactions. Typically, in these devices, the fuel molecules are oxidized at the anode, and the oxidant is reduced at the cathode [1]. Based on this concept, several types of fuel cells exist that operate using various types of fuels [2]. Among them, H_2_-based fuel cells are widely used for high-energy transportation applications, and several H_2_ fuel cell-based electric vehicles are already available in the commercial market [3]. However, the widespread use of H_2_-based fuel cells in the consumer market still faces several challenges due to the high cost of Pt-based catalysts and problems associated with H_2_ production, storage, and transportation [4,5,6,7]. Moreover, H_2_-based fuel cells are not suitable for low-power portable applications. In contrast, fuel cells that operate with liquid fuels at low or ambient temperatures offer several advantages, such as easier fuel production, storage, and transportation. Among the liquid-based fuel cells, direct alcohol fuel cells (DAFCs) have attracted special attention due to the fact that their ease of oxidation and reduction potentials are similar to those of H_2_-based fuel cells.

**Figure 1:**
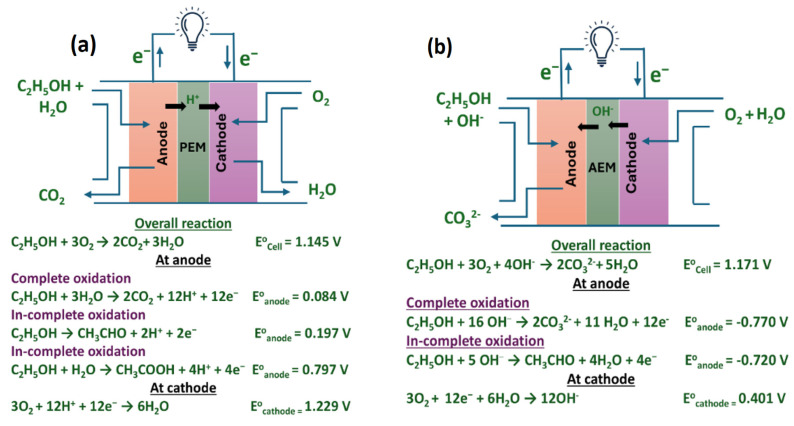
Operating principles of direct methanol fuel cells in (**a**) acid and (**b**) alkaline conditions.

**Figure 2:**
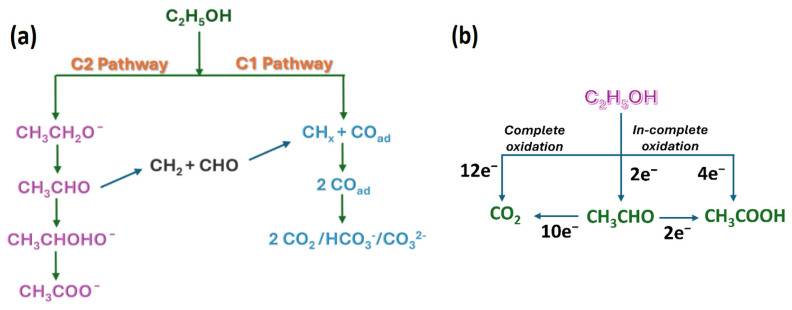
(**a**) Methanol electro-oxidation pathway, and (**b**) schematic representation of complete and incomplete oxidation pathways of ethanol.

**Figure 3:**
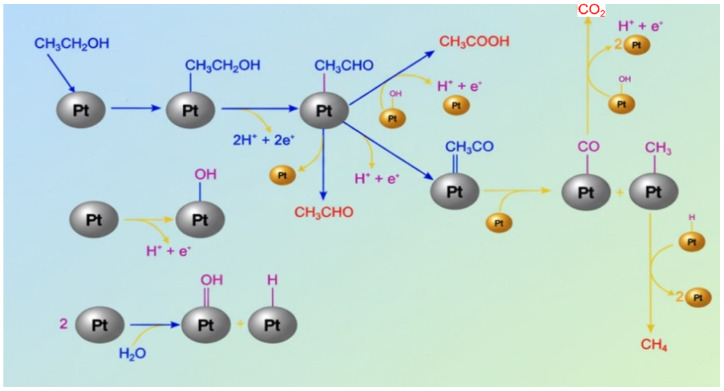
Mechanism of ethanol electro-oxidation over a Pt electrocatalyst. Obtained from Ref. [8], open access.

Direct methanol fuel cells (DMFCs) and direct ethanol fuel cells (DEFCs) are two important alcohol-based fuel cells that have gained considerable interest because of their high volumetric energy densities compared with compressed gaseous H_2_ fuels. The theoretical energy required to compress hydrogen isothermally from 20 bar to 350 bar (5000 psi or ~35 MPa) is 1.05 kWh kg^−1^ H_2_, and that required for compression to 700 bar (10,000 psi or ~70 MPa) is 1.36 kWh kg^−1^ H_2_. By comparison, the volumetric energy densities of CH_3_OH and C_2_H_5_OH are 4.82 and 6.28 kWh L^−1^, respectively [8]. In addition, compared to the mass of H_2_ in storage (in the form of compressed H_2_ in a gaseous cylinder or storage in the form of metal hydrides with 4–5 wt.%), the alcohols have much higher gravimetric densities (H_2_: 1.2 kWh kg^−1^; CH_3_OH: 6.1 kWh kg^−1^; C_2_H_5_OH: 8.0 kWh kg^−1^). Between the two, C_2_H_5_OH-based fuel cells have gained particular interest, largely for their renewable nature, lower toxicity, and low fuel crossover issues compared to methanol [9]. Although C_2_H_5_OH has high gravimetric and volumetric energy densities, several critical issues still hinder the commercialization of DAFCs. Ethanol is a larger molecule than H_2_, and therefore the ethanol oxidation reaction (EOR) requires a higher activation energy. Due to the structural complexity of ethanol involving C-C bonds, the EOR requires multiple electron transfer pathways. While the complete oxidation of ethanol to CO_2_ requires 12 electron transfer processes, also accompanied by C-C bond cleavage, partial oxidation, typically involving 2 or 4 e^−^, leads to the formation of intermediates such as acetaldehyde or acetic acid (Figure 1, Figure 2 and Figure 3). In practical conditions, it is not possible for ethanol to convert into CO_2_ due to poor, highly sluggish oxidation kinetics and strong C-C bonds, which lead to the formation of various intermediate oxidation products. Therefore, the kinetics of the EOR are one of the fundamental issues with DEFCs that require a highly active electrocatalyst [10,11].

The basic operating principle of DEFCs involves the anodic oxidation of ethanol to generate electrons and the cathodic oxygen reduction reaction (ORR), in which O_2_ is reduced to water. Furthermore, ethanol oxidation can occur in both acidic and alkaline electrolytes, although the reaction mechanisms differ, while the overall reaction products remain the same.

### 1.1. EOR and Anode Electrocatalysts in Acidic DEFCs

In acidic-type DEFCs, ethanol oxidation occurs at relatively low pH values, and the membrane electrode assemblies (MEAs) generally contain proton-exchange membranes (PEMs) such as Nafion 115, Nafion 117, or other hydrocarbon-based polymers [12,13]. During DEFC operation, fuel (ethanol+H_2_SO_4_) is reacted; therefore, the concentration of ethanol decreases over time, and hence it is essential to replenish the anolyte with fresh electrolyte. In acidic DEFCs, Pt-based catalysts have been widely used as efficient electrocatalysts for the EOR. However, the poisoning of Pt catalysts by adsorbed CO species during the EOR remains one of the important drawbacks. To mitigate CO poisoning, the introduction of secondary oxophilic metals, such as Ru or Sn, has been found to be essential. These metals promote a bifunctional mechanism by providing oxygen-containing species, such as –OH groups, at Ru or Sn sites, thereby helping to overcome Pt poisoning [14,15,16]. Furthermore, the electrocatalytic activity of Pt-based catalysts also depends on structure, morphology, and particle size [17,18]. The EOR involves not only dehydrogenation reactions but also requires the cleavage of C-C bonds in order to completely oxidize to CO_2_ [19].

### 1.2. EOR and Anode Electrocatalysts in Alkaline DEFCs

In alkaline-type DEFCs, the oxidation of fuel (ethanol+NaOH) occurs at a relatively higher pH (between 8 and 12) and the MEAs use anion-exchange membranes. Alkaline DEFCs offer advantages over acidic DEFCs due to faster EOR and ORR kinetics in alkaline pH. In alkaline electrolytes, non-platinum-type catalysts have shown better catalytic activity in alkaline media compared to conventional platinum-based catalysts, both on cathode and anode sides [20]. Therefore, several inexpensive noble metals such as palladium-, gold- and silver-based catalysts can be used for the EOR, and various non-precious metal catalysts can be used as ORR catalysts at the cathode sides, significantly decreasing the overall cost of the fuel cells [21,22]. Compared with acid media, the less corrosive environment of alkaline media allows the use of a wider range of metals as catalysts. Figure 1 shows the pictorial representation of the working principles of DEFCs in acidic and alkaline electrolytes and their electronic mail reactions.

### 1.3. Mechanism of EOR

Ethanol (EtOH) electro-oxidation is generally believed to proceed through two distinct pathways, namely the C_1_ and C_2_ paths, which are distinguished by whether cleavage of the C-C bond occurs. In the C_1_ pathway, the C-C bond breaks, whereas in the C_2_ path it does not, leading to the formation of different types of reaction products as shown in Figure 2. Ideally, complete EtOH oxidation should involve the transfer of 12 electrons. However, most electrocatalysts are unable to completely oxidize EtOH to CO_2_. Therefore, three major products are typically formed: CH_3_CHO through a two-electron process, CH_3_COOH through a four-electron process, and CO_2_ through a 12-electron process. Among these, acetic acid is the most commonly formed product in the case of multimetallic Pt-based catalysts because its formation requires lower energy than CO_2_ formation [23].

### 1.4. EOR over Pt Surface in Acidic Electrolytes

The EOR on a Pt surface proceeds via sluggish, complex and multistep reactions as follows. Although the complete reaction mechanism remains under debate, a common conclusion has been made by researchers indicating that acetaldehyde and acetic acid are the major byproducts of the EOR over Pt-based catalysts in acidic electrolytes. During the EOR, CO intermediate (Pt-CO_ads_) formation is said to block the Pt surface and requires a potential of >0.6 V to get converted to CO_2_ via water oxidation, producing Pt-OH and Pt-O species [8]. A detailed EOR mechanism on the Pt surface has been given in Figure 3.

The adsorbed Pt-CO_ads_ reduces the availability of the catalytic active sites, highlighting the importance of -OH groups in playing a critical role in the oxidation of EtOH. The presence of a second oxophilic metal that provides continuous adsorbed -OH species is paramount. Several oxophilic metals, such as Pd, Ru, Au, and Ni, have been found to assist in the conversion of Pt-CO_ads_ into CO_2_ by contributing -OH species, as indicated in the below equations (Equations (1) and (2)) [24,25,26].

M + H_2_O → M-OH + H^+^ + e^−^ (1)

Pt-CO + M-OH → Pt + M + CO_2_ + H^+^ + e^−^ (2)

### 1.5. EOR over Pd Surface in Alkaline Electrolytes

EtOH oxidation on Pd-based catalysts has been reported to exhibit superior electrocatalytic activity to that of benchmark Pt-based catalysts in an alkaline environment. Other elements, such as Au and Ag, have also been found to exhibit high electrocatalytic activity toward the EOR. The EOR mechanism on Pd and alloy (Pd-M) surfaces has been given in the below equations (Equations (3)–(11)).

Ethanol electro-oxidation on Pd-based catalyst

Pd + OH^−^ → Pd-OH_ads_ + e^−^ (3)

Pd + CH_3_CH_2_OH → Pd(CH_3_CH_2_OH)_ads_ + M + CO_2_ + H^+^ + e^−^ (4)

Pd(CH_3_CH_2_OH)_ads_ + 3OH^−^ → Pd(CH_3_CO)_ads_ + 3H_2_O + 3e^−^ (5)

Pd (CH_3_CO)_ads_ + Pd-OH_ads_ + OH^−^ → 2Pd + CH_3_COO^−^ + H_2_O (6)

Ethanol electro-oxidation on Pd-M alloy-based catalyst

Pd-M + C_2_H_5_OH → Pd-M-(C_2_H_5_OH) (7)

Pd-M-(C_2_H_5_OH) + 3OH^−^ → Pd-M-(CH_3_CO)_ads_ + 3H_2_O + 3e^−^ (8)

Pd-M + OH^−^ → Pd-M-OH_ads_ + e^−^ (9)

Pd-M-(CH_3_CO)_ads_ + Pd-M-OH_ads_ → 2Pd + 2M + CH_3_COOH (10)

CH_3_COOH + OH^−^ → CH_3_COO^−^ + H_2_O (11)

### 1.6. Anode Electrocatalysts for EOR and Prerequisites

DEFCs operate through an anodic EOR and cathodic ORR. Both reaction kinetics significantly affect the overall fuel cell performance. Due to diverse reactants on anodes and cathodes, different catalyst systems and catalyst designs are essential since both half-cell reactions are different from each other. For efficient anode electrocatalysts, there are three general prerequisites: (i) a high exposure of catalytic active sites for efficient cleavage of C-C bond in EtOH; (ii) selectivity of the anode electrocatalyst for the complete conversion of EtOH into CO_2_ and H_2_O; and (iii) an efficient oxophilic, bifunctional metal and an active metal that gives -OH groups for catalytic cycle efficiency by removing the CO_ads_ and CH_x_ intermediates. In addition to this, catalyst morphology, shape, and composition also affect electrocatalytic activity [20].

The efficiency of DEFCs is highly dependent on the complete oxidation vs. incomplete oxidation of EtOH, which produces either CO_2_ or partial oxidation products such as acetaldehyde and acetic acid [27]. Pt, Pd and their alloys supported on high-surface-area carbon have been considered as state-of-the-art catalysts for the EOR. Among the most investigated catalysts, Pt-Sn has shown excellent catalytic performance, particularly at an atomic ratio of 3:1 [28,29]. However, the high cost of Pt has been a concern for the commercialization of DEFCs, and hence efforts have been made to reduce Pt usage by alloying with cost-effective noble metals and non-precious metals [30]. Accordingly, several bimetallic and trimetallic alloy catalysts supported on carbons have been proposed for the EOR [31]. In addition, novel synthesis strategies have been proposed to build advanced electrocatalysts from bimetallic and trimetallic alloy catalysts in the form of core–shell nanoparticles, nanowires, hollow spheres, and dendritic structures, which have been projected as excellent electrocatalysts for advanced EORs.

Most monometallic, bimetallic and trimetallic catalysts utilize high-surface-area carbon as a support material to disperse metallic active sites [32]. Support materials play a critical role in the heterogeneous electrocatalysts. The main prerequisites of the support materials include (a) a high electronic conductivity, (b) high surface area and porosity, (c) high graphitic nature, (d) strong metal–support interaction, (e) resistance to carbon corrosion under acidic and highly alkaline solutions, and (f) structural and mechanical integrity [33]. In general, Vulcan carbon or carbon black has been widely used as a carbon support in fuel cell electrocatalysis [34]. However, it is well documented that Vulcan carbon or carbon black is highly sensitive to electrochemical carbon corrosion under highly acidic/alkaline conditions [35]. This issue is relevant to both the anodes and cathodes of DEFCs. On the anode side, EtOH is mixed with either an acid or a base, whereas, on the cathode side, the highly oxidizing environment caused by O_2_ aggravates carbon corrosion. This consequently affects the stability and degradation of both anode and cathode catalysts. Carbon supports generally act as platforms to host nanoparticles and facilitate electron transfer during electrochemical reactions [36]. Electrochemical carbon corrosion means the conversion of solid carbon support into gaseous CO_2_. When the physical carbon is lost, the supported nanoparticles detach from the carbon support and are either combined with the other nanoparticles to form aggregates or are detached and wash away; both lead to a loss of metallic active sites and electrochemical active surface area (ECSA) [37]. Furthermore, traditional Vulcan carbon or carbon black has poor graphitic domains, and the high density of surface oxygen functionalities leads to poor stability of the electrocatalysts. In this regard, support modification is found to be an effective strategy to improve the stability of the catalyst. Among several modifications, the surface functionalization of carbon supports with polymers, using heteroatom-doped carbons with particles such as N, P, S, and F doped to the carbon, has been found to be effective in mitigating carbon corrosion [38]. On the other hand, various other highly graphitized carbon supports have also been explored as excellent and stable carbon supports, such as carbon nanofibers, carbon nanotubes, highly graphitic carbons, graphene, etc. [39]. On the other hand, non-carbon supports based on metal oxides have also been employed as support materials for electrocatalytic applications [40,41]. Several metal oxides have been explored, such as SnO_2_, TiO_2_, MoO_3_ and Maxene’s, etc. [41,42,43]. However, metal oxide-supported catalysts suffer from low surface areas and poor electronic conductivity.

**Figure 4:**
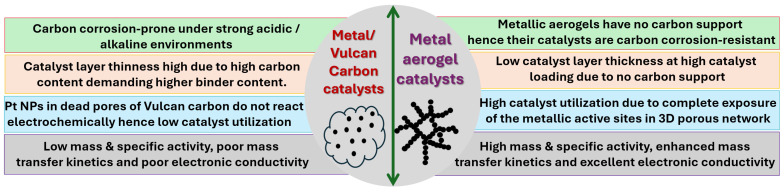
Advantages of metal aerogel electrocatalysts over traditional carbon-supported catalysts.

On the other hand, self-supported, all-metallic aerogel catalysts have emerged as novel catalyst materials that serve as both metallic active sites for the EOR and also support materials (Figure 4).

Metal aerogels possess several advantages (Figure 4), such as being completely made out of a metallic backbone; they exhibit excellent electrical conductivity and a high porosity and mechanical stability [44]. When compared to traditional carbon-supported catalysts, self-sustained metal aerogel catalysts possess several other advantages, such as the following:

(i) No carbon—No support corrosion: Self-supported metal aerogel catalysts are entirely made of a metallic backbone; therefore, catalyst instability arising from carbon support corrosion can be completely avoided. Furthermore, the complete metallic network with a direct metal–metal bond fastens electron transport and hence enhances the electrocatalytic activity through faster electron transport to/from the adsorbate reactants.
(ii) High metallic active site utilization: Unlike conventional carbon black or Vulcan carbon, which contains dead pores in which metallic nanoparticles may be deeply buried and inaccessible for electrochemical reactions, metal aerogels process a three-dimensional network with high porosity. This structure makes more metallic active sites accessible to reactants and thus greatly enhances catalyst utilization.
(iii) Simple synthesis process: Metal aerogel catalysts are synthesized through the reduction of metallic precursors with the help of reducing agents, which generally requires no further treatment. This is advantageous compared with certain catalyst synthesis methods that require heat treatment or special post-synthesis procedures to remove ligands, surfactants, polymers, or structure-directing agents, which can lengthen and complicate the synthesis process.
(iv) Reduced catalyst layer thickness: In MEAs, self-supported catalysts significantly reduce the catalyst layer thickness. When applying at the cathodes, traditional carbon-supported catalysts contribute a very high catalyst layer thickness due to the contribution from the carbon vs. the actual metallic active sites. In contrast, metal aerogels are completely made of metallic components; therefore, they can significantly reduce the catalyst layer thickness and enhance reactant utilization.

## 2. Metal Aerogels and Their Synthesis

Metal aerogels are a class of 3D porous metallic networks that are built entirely from metallic atoms. Metal aerogels are hybrid materials that inherit the properties of metals (such as electrical conductivity, mechanical strength, electrocatalytic activity) and aerogels (such as a 3D porous nature, large specific surface area). Discovered in 2009 by Prof. Eychmüller and Leventis’s group [45,46], metal aerogel catalysts find several applications in electrocatalysis, such as in oxygen reduction reactions, methanol oxidation reactions, hydrogen evolution reactions, and oxygen evolution reactions [47]. The first Pd metal aerogel as a high performance electrocatalyst for the EOR was uncovered in 2012 [48]. Since then, metal aerogel synthesis has been extended to several metals such as Au, Pt, Pd, Ag, Ru, Rh, Cu, Ni and Os [49].

**Figure 5:**
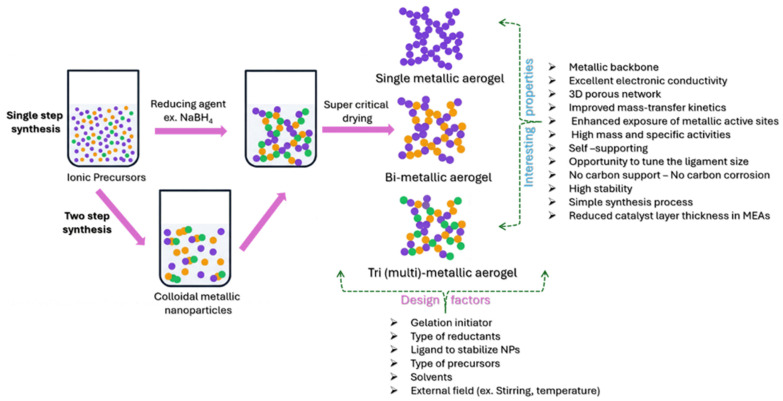
Typical synthesis process, interesting properties, and design factors of metal aerogels.

Briefly, metal aerogels are derived from a “bottom-up” sol–gel synthesis by reducing metal ionic precursors into metals, and the obtained metallic product is afterward dried either by freeze-drying or supercritical drying [50] (Figure 5). Unlike conventional drying in a hot-air oven that collapses the structural integrity and porosity of the product (resulting in xerogels), freeze-drying or supercritical drying preserves the intrinsic porous network inherited from the initial product [51]. The sol–gel synthesis of metal aerogels comprises three steps: (i) the reduction of metallic precursors into metal atom colloidal “sol” NPs; (ii) the conversion of “sol” into “gel”, either by destabilizing the sol NPs or by spontaneous aging (gelation) of the solution to finally obtain the “hydrogel”; and (iii) obtaining the metal aerogel through supercritical drying/freeze-drying [52].

Obtaining a wet hydrogel (branched nanowires are the building blocks) through the self-assembly of nanoparticles via gelation is an important and critical step in the synthesis of metal aerogels. The synthesis processes of metal aerogels can be broadly classified into two categories: (i) a two-step synthesis route and (ii) a single-step synthesis route [53]. In two-step synthesis routes, first, colloidal nanoparticles are obtained through the addition of reductants to the metal precursor solutions, which are then allowed to form a monolith gel by adding destabilizing agents (ethanol, H_2_O_2_, salts, ligand exchange, heating, etc.) deliberately to induce gelation [54,55]. In the one-step synthesis process, hydrogels are spontaneously generated through the in situ reduction of noble metal precursors using NaBH_4_ as a reductant, without the prior production of suitably stabilized nanoparticles. In a typical synthesis, for a solution containing precursor metal ions, aqueous NaBH_4_ is added, during which metallic ions (M^n+^) are converted to metallic atoms (M^0^). The metallic atoms are then nucleated into clusters. Due to the high surface energy of metallic atoms and clusters, several nanoclusters attach, forming interconnected-chain structures. The continuous crosslinking of the metallic chains results in a metal hydrogel. Based on several studies, it is clear that colloidal NPs self-assemble into short nanowires, small branched networks, and finally largescale interconnected nanowire networks in a possible and widely accepted mechanism for the formation of metal aerogels with 3D, porous, interconnected nanowire networks. Furthermore, the NaBH_4_ functions only as a reducing agent at lower concentrations (R/M < 2). However, at higher concentrations (R/M < 50), after reducing all metallic precursors, the excess NaBH_4_ decomposes products such as BH_4_^−^ or BO_2_^−^, essentially acting as a stabilizer, in addition to the role of reductant. With an even further increase in concentration (R/M > 50), the NaBH_4_ has a salting-out function in addition to being a reductant and stabilizer. At higher concentrations, the NaBH_4_ acts as an electrolyte by initiating nanoparticle aggregation and fusion in the presence of in situ-formed decomposed products of NaBH_4_ into BH_4_^−^ or BO_2_^−^. The salting-out function of excessive NaBH_4_ naturally acts as a destabilizing agent to fasten the gelation of the NPs into hydrogel. In addition, NabH_4_ decomposition also releases H_2_ gas, which helps move and lift the metallic nanoparticles so that they can fuse and attach to each other to produce the hydrogels. Also, side products such as BO_2_^−^/B(OH)_4_^−^ increase the solution pH and ionic strength, which also promotes aggregation and hence gel formation. The detailed effects of the concentration of NaBH_4_ on reduction have been explained in the subsequent sections. 

Metal aerogel-based catalysts have gained tremendous interest in recent years, especially for electrocatalytic applications, due to their self-supporting nanoparticle morphology, with a continuous metallic skeleton that results in enhanced electron transport across the 3D hierarchical structure. Due to their unique features that distinguish them from traditional carbon-supported catalysts, metal aerogels are also very different from carbon aerogels and fundamentally different from other electrocatalyst such as alloy, core–shell, nanoframe and other porous catalyst-based systems. Metal aerogel synthesis comes with the great advantage of a simple and one-pot synthesis strategy, not requiring special post-synthesis steps, making it unique, as it can expose abundant active sites to cleavage of the C-C bond and improved mass transport properties. Most of the recent reviews focus on nanoparticle-based electrocatalysts but not metal aerogel-based catalysts [11,15,16,21,24]. Due to the rapidly evolving research interest on metal aerogels for electrocatalysis, a review of the recent advances in metal aerogels in terms of synthesis and electrocatalytic activity is urgently required. Recent review articles on metal aerogels mostly focus on synthesis aspects, and reviews on electrocatalytic applications, especially for the EOR, are not reported so far.

**Figure 6:**
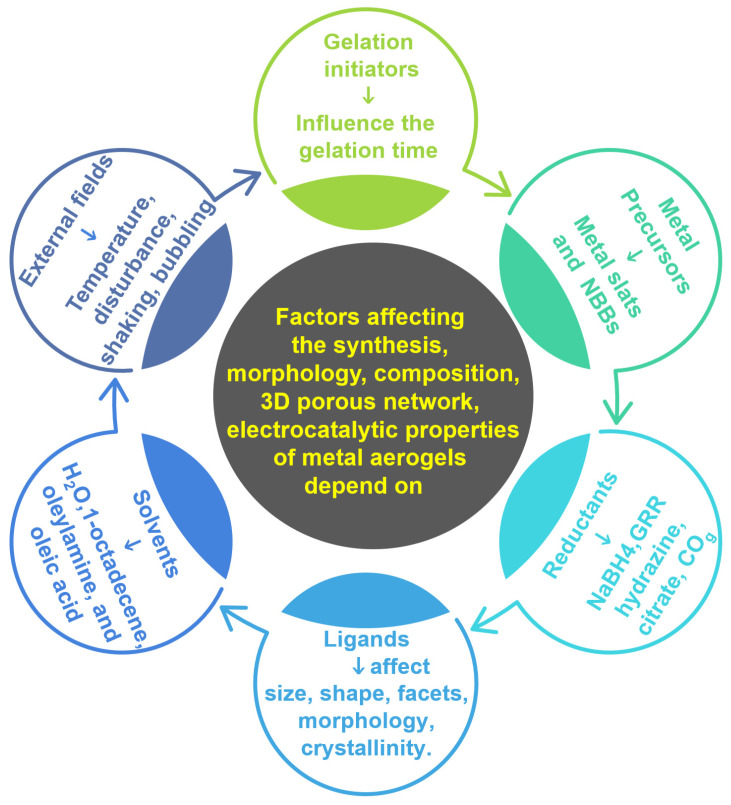
Factors affecting synthesis, morphology, composition, 3D porous network, and ethanol electro-oxidation activity of metal aerogels.

A variety of metal aerogels were synthesis by using two-step and single-step synthesis strategies [53]. The morphology, composition, electrocatalytic properties, and 3D porous network of metal aerogels are strongly dependent on the synthesis process. There are six important design factors that must be considered carefully during metal aerogel synthesis:

(1) Initiators: These are the agents that induce the gelation and hence strongly affect gel formation and the gelation time.
(2) Precursors: The type of precursors influence the metal aerogel’s morphology; the precursors could mostly be metal salts in some cases, nano building blocks, or metal gels.
(3) Reductants: Since most metal aerogel synthesis utilizes metal salts as precursors, the reductants play an important role in converting ionic metal precursors to zero-valent metallic atoms that further self-assemble into a 3D gel network. The most common reductants involve liquid-phase reduction using NaBH_4_, hydrazine, etc. Solid–solid reduction at high temperatures and liquid–gas reduction methods are also explored.
(4) Ligands: The interaction between ligands and nanoparticles affects the size, shape, morphology, crystallinity and facet of the obtained metal aerogels. Several researchers used a variety of ligands such as CO gas, citrate, cyclodextrins, thiol-containing glutamic acid, etc. However, one of the persistent problems of using ligands is their shielding effect on nanoparticles; limited techniques are available to completely remove them from the nanoparticle surface. Recently, utilizing excessive NaBH_4_, which acts as both a redundant and a stabilizer, has mostly solved the issue of ligand blocking by organic molecules.
(5) Solvents: The solvents play an important role in the even dispersion of metallic salt precursors and reductants. For a majority of the metal aerogels, H_2_O is the best solvent. Other non-polar solvents such as 1-octadecene, oleylamine, and oleic acid have also been used.
(6) External fields: Apart from all the five major factors, external fields also affect metal aerogel synthesis, especially the gelation process. Temperature, increasing concentration by raising the precursor amount, counterintuitive disturbance, shaking and bubbling are some of the external force fields that are investigated (Figure 6).
  Detailed information on metal aerogel synthesis methods, factors affecting gel formation, the mechanism of gelation, background on the theoretical and experimental aspects of gelation, and the properties of metal aerogels have been discussed in recent reviews by none other than the discoverer of metal aerogels, Prof. Alexander Eychmuller, and other pioneering researchers of metal aerogels. Therefore, we choose to exclude a discussion in this review and ask the readers to refer to the following literature for more information [53,56,57,58,59,60,61].

In this review, we overviewed, summarized and critically analyzed the use of metal aerogel catalysts as efficient anode catalysts for EORs in DEFCs. Various synthesis routes, structure–property relationships and their function as anode electrocatalysts have been critically reviewed. Due to their 3D porous metallic nature, noble meal aerogel catalysts were found to exhibit excellent ethanol oxidation currents, anti-poisoning for reaction intermediates, a high mass, and specific activities of 5–20 times that of traditional Pd/C catalysts. In conclusion, it is shown that metal aerogel catalysts exhibit enhanced activity for ethanol electro-oxidation, with higher oxidation currents over traditional Pd/C catalysts. Despite this, in order to truly realize the commercial applications of metal aerogels, several challenges have been clearly and elaborately stated as future perspectives and research directions in the field of metal aerogel electrocatalysis.

### 2.1. Metal Aerogels as Electrocatalyst for Ethanol Oxidation Reaction

#### 2.1.1. Monometallic Pd-Based Metal Aerogels

**Figure 7:**
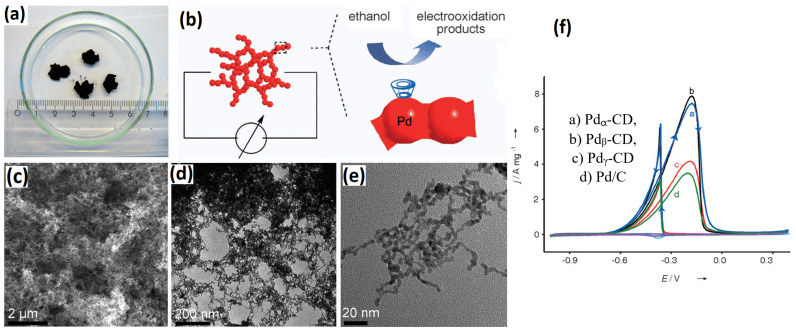
(**a**) Picture of Pd aerogel powder; (**b**) schematic of cyclodextrin-modified palladium aerogel; (**c**) SEM and (**d**,**e**) TEM images of Pd aerogel; (**f**) ethanol electro-oxidation curves of various catalysts. Reproduced with permission from Ref. [48].

Palladium (Pd)-based metal aerogels are by far the most studied electrocatalysts for the EOR. Pd is recognized for its high ethanol electro-oxidation activity in alkaline solutions [62]. In 2012, Prof. Alexander Eychmuller introduced the Pd aerogel and its application as an EOR electrocatalyst [48]. This opened a new avenue for advanced nanostructures of noble metals as possible electrocatalysts for liquid fuel cells. The Pd aerogels were initially synthesized by using cyclodextrins as stabilizers, NaBH_4_ as a reductant, and supercritical drying. Host–guest interactions between cyclic oligosaccharides, specifically cyclodextrins (CDs), and metal nanoparticles provide an effective strategy for controlling nanoparticle size. Among the three commonly studied cyclodextrins—α-, β-, and γ-CD—β-cyclodextrin-based palladium (Pd_β-CD_) systems demonstrate superior performance. In particular, Pd_β-CD_ exhibits enhanced porosity, improved crystallinity, and significantly higher electrocatalytic activity toward the ethanol oxidation reaction. The Pd_β-CD_ hydrogel exhibits a highly porous and interconnected 3D network of Pd atoms in the form of nanowires, with ultrathin nanowires with a diameter of 3.6 nm. Pd aerogel was characterized using X-ray photo electron spectroscopy, FT-IR spectroscopy, energy-dispersive X-ray spectroscopy, and thermogravimetry analysis. These analyses indicated that the Pd_β-CD_ consists of two main components (about 56 wt.% of Pd and about 44 wt.% of β-CD). The TEM morphologies showed interconnected and fused nanowires of Pd forming branched structures. The mechanism of Pd_β-CD_ involves four distinct stages: In the first stage, the metallic precursors are reduced to zero-valent Pd atoms that grow into nanoparticles. In the second stage, the nanoparticles are slowly fused to form short Pd nanochains. In the third stage, the short Pd nanochains interconnect with other Pd nanochains into 3D interpenetrating networks of Pd chains that eventually grow into the Pd hydrogel. Though β-CD was used as stabilizer to cap the Pd nanoparticles due to its large organic molecular structure, which controls and slows down the nanoparticle self-assembly via steric hindrance, the control experiment proved that Pd nanoparticles self-assemble even without the need of stabilizers. This conclusion is so important, as this simple process can be extended from Pd to other noble metals and non-noble metals in the periodic table; it opened up a new avenue of advanced materials for electrocatalytic applications in the field of metal aerogels. When Pd_β-CD_ was used as an electrocatalyst for the EOR and compared with traditional Pd/C catalysts, surprisingly, the Pd_β-CD_ exhibited a similar characteristic of two well-defined current peaks in the electro-oxidation of alcohol in 1 M KOH + 1 M EtOH. The maximum current during the forward scan is associated with the oxidation of newly chemisorbed species produced through EtOH adsorption. The significant anodic oxidation current observed during the reverse scan signifies the removal of carbonaceous intermediate species that were not completely oxidized in the forward scan. In general, the peak current at forward and backward scan (I_f_/I_b_) is taken as a tool to classify the superiority of the catalysts [20]. A high I_f_/I_b_ ratio generally represents a high activity and efficient oxidation in the anodic forward scan. The highest I_f_/I_b_ for Pd_α-CD_ was found to be 1.28, indicating more efficient EtOH electro-oxidation and a lesser poisoning. The loading of Pd on the glassy carbon electrode was limited to 20 μg cm^−2^. In addition to the high I_f_/I_b_, Pd_α-CD_ exhibited a better onset potential compared to the Pd/C catalyst and a higher mass activity, at about 2.3 times higher than the Pd/C catalyst (10 wt.%) (Figure 7).

**Figure 8:**
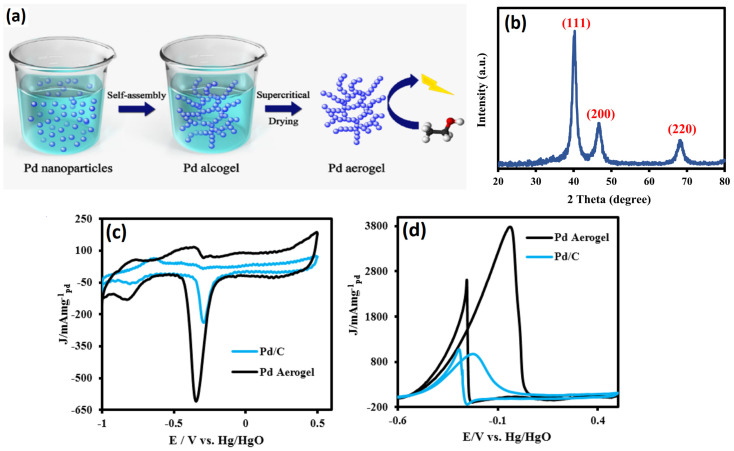
(**a**) Schematic of Pd aerogel synthesis; (**b**) X-ray diffraction patterns of Pd aerogel; (**c**) CV curves and (**d**) ethanol oxidation curves of Pd/C and Pd aerogel. Reproduced with permission from Ref. [66].

The formation of hydrogels is a key step in the synthesis of aerogels via self-assembly and gelation. This gelation was achieved in several ways: by adding salts like Ca^2+^ ions [55]; adding NaBH_4_ to the reactants at higher/lower temperatures [63]; and adding gelating agents like hydrazine monohydrate [64]. A study by Douk et al. [49] employed sodium carbonate in glyoxylic acid monohydrate as a unique reducing agent to synthesize Pd aerogel. A comprehensive analysis of the influence of the sodium carbonate-to-glyoxylic acid ratio on the gelation time has been conducted. The porous Pd 3D network was achieved through a controlled reduction and self-assembly of the reduced Pd atoms. The gelation of Pd aerogel is significantly influenced by the variation of sodium carbonate, as an increase in carbonate concentration enhances the gelation process of Pd aerogel while maintaining a constant concentration of glyoxylic acid. With a change in the ratio of sodium carbonate:glyoxylic acid from 1:1 to 1:7, the gelation time was shortened from 245 min to 60 min. The optimum conditions for the gelation were found at 1:6. The obtained Pd aerogel exhibited excellent crystallinity, interconnection and interpenetration of Pd atoms into the 3D network. The N_2_ adsorption/desorption isotherm of Pd aerogel shows the mesopores that help in the easier accessibility of the reactants and removal of the products. The onset potential of Pd aerogel for the oxidation of ethanol is found to be 82 mV higher than the Pd/C catalyst. In the alkaline ethanol reduction reaction, Pd aerogel showed an excellent mass activity of 4703 mA mg^−1^_Pd_, whereas Pd/C exhibited a low electrocatalytic activity of 1007.9 mA mg^−1^_Pd_ mass activity. These results indicate that Pd aerogel shows excellent ethanol oxidation electrocatalytic activity. The I_f_/I_b_ for Pd aerogel is found to be higher (1.59) than the Pd/C catalyst (0.6), indicating that, on the Pd aerogel surface, it is easier to remove the incompletely oxidized intermediate products [65]. In another study [66], Pd aerogel was synthesized by using an unconventional ethanolic–sodium hydroxide (C_2_H_5_-OH and NaOH) as a reducing agent, and the Pd aerogel was synthesized in a single step via a supercritical drying process with CO_2_. During the reduction process, the Pd NPs formed initially then defused together into Pd nanochains networks. The effective combination of hydrogen bonds and van der Waals forces is responsible for the formation of porous nanochain networks. The fusion of NPs is the result of Ostwald ripening, which uses both intermolecular forces to promote the oriented attachment of Pd nanocrystalline structures [67]. The ethanol in the pores of the palladium alcogel was replaced with acetone before the supercritical drying process began. After 72 h of submersion in acetone, the palladium alcogel was removed. In the end, the alcogel was transformed into aerogel by drying the wet-gel structure that had been exchanged with acetone using supercritical CO_2_. The XRD studies revealed sharp Pd diffraction peaks at the 2θ values of 40.1, 46.7, and 68.3°, corresponding to Pd (111), Pd (200), and Pd (220) diffraction peaks, confirming the crystalline peaks. Most importantly, the presence of Pd with a dominant (111) crystallographic plane is believed to enhance ethanol adsorption and oxidation activity due to its resistance to oxidation during the electrochemical reaction [66]. Interestingly, the PD aerogel synthesized via ethanolic sodium hydroxide (C_2_H_5_-OH and NaOH) shows a greater proportion and content of Pd (111), and therefore it is expected that the Pd aerogel presents higher EOR performance. The TEM images show the highly interconnected Pd nanochains fused together into a porous network. The interconnected particles are essential for the continuous movement of electrons during the electrochemical reaction. In supported catalysts such as Pd/C, electrons must transfer from the carbon support to the metal active sites and then to the reactants. However, carbon supports often contain both conductive (sp^2^) and less conductive (sp^3^) carbon, which can create non-uniform pathways and increase electron transport resistance. In contrast, metal aerogels consist of a continuous metallic network and metallic conductivity, where electrons can move more directly through interconnected metal atoms with lower resistance. This leads to more efficient electron transfer during the reaction, which enhances electrocatalytic performance. When applied as an anode catalyst for the EOR, the Pd aerogel catalyst exhibited a higher mass activity of 3.5 times that of Pd/C catalyst. In addition, the Pd aerogel also exhibited a higher I_f_/I_b_ ration than the traditional Pd/C catalyst, significantly implying that the Pd aerogel catalyst can enhance the EOR and has a mass activity suitable for commercial applications (Figure 8).

#### 2.1.2. Monometallic Pd Metallene Aerogels

**Figure 9:**
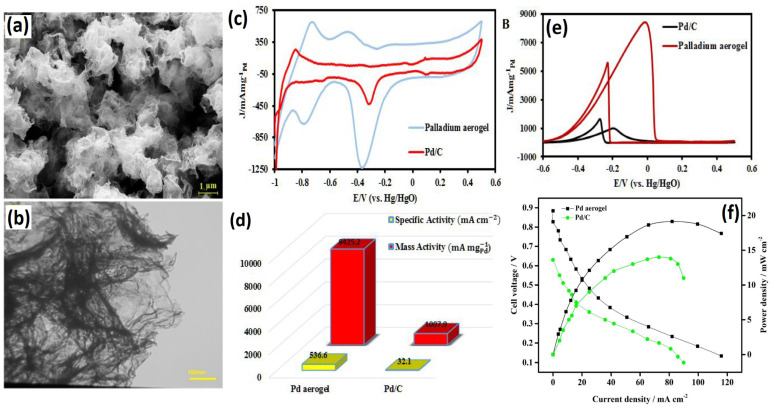
(**a**) SEM and (**b**) TEM images of Pd metallene catalyst; (**c**) CV curves of Pd aerogel and Pd/C catalysts; (**d**) mass activity and specific activity values of Pd aerogel and Pd/C catalysts; (**e**) ethanol oxidation curves recorded by cyclic voltammetry; (**f**) fuel cell polarization curves of Pd metallene and Pd/C catalysts. Reproduced with permission from Ref. [73].

Recently, nanostructured two-dimensional materials have been gaining a lot of interest in the field of electrocatalysis. The 2D materials are known to possess large surface areas coupled with a high density of exposable active sites, excellent during electronic electrochemical reactions [68]. In this regard, 2D metallic aerogels in the form of nanosheets known as “metallenes” have become popular in the electrocatalysis field. Metallenes are atomically thin two-dimensional structures made of pure metallic elements that have a variety of metal active sites, high conductivity, and atomic utilization [69]. Metallenes have inherent strain associated with their specific thickness and curvature, which significantly affects the surface electronic structure and energetics of reaction intermediates, thereby enhancing their catalytic activities [70]. To date, there are only a limited number of studies focused on the synthesis of Pd metallenes. In addition, the general process of synthesizing metal aerogels includes the addition of reducing agents to the metallic precursors followed by supercritical drying. Metallene synthesis utilizes structural directing surfactants and CO gas, which can adjust the Pd seeds’ lateral growth rate by combining with the (100) planes [71,72]. By employing surfactant-induced Pd nanoparticle self-assembly into 2D Pd sheets, Douk et al. [73] synthesized Pd metallene, with the Pd hydrogel synthesized via CO-butyric acid-containing Pd(acac)_2_ in the presence of CTAB. In order to overcome isotropic electrostatic repulsions, the colloidal suspension was heated to a point where gelation could begin. To create a monolithic 3D superstructure of palladium aerogel, the solvent was extracted from the 3D gel’s pores using supercritical drying. In this synthesis process, CO gas serves as a ligand and reducing agent that also facilitates effective anisotropic growth to obtain Pd NSs, unlike the traditional liquid-based reductants [74]. Meanwhile, butyric acid acts as a solvent and capping agent. During the reduction process, CO gas is adsorbed on the preformed Pd atoms that grow on the Pd (111) phase, with BA acting as a capping agent; CTAB accelerates the Pd NPs fusing into Pd nanosheets [75]. Effective gelation and anisotropic conditions are created by enhancing the temperature by 55 °C, which acts as a destabilizer factor in the 3D hydrogel, which then undergoes freeze-drying to obtain the low-density Pd aerogel metallene. The as-obtained Pd metallene showed a 3D network of expanded NSs with plentiful open pores. The Pd metallene obtained in this way showed an excellent electrochemical surface area, high electrocatalytic activity and enhanced mass activity compared to the Pd/C catalyst in a traditional three-electrode system. The I_f_/I_b_ of the Pd metallene was found to be 1.48, whereas it was 0.6 for Pd/C, ascertaining the excellent electrocatalytic activity of the catalyst, in addition to the high EOR onset potential of the Pd metallene electrocatalyst. Surprisingly, the Pd metallene catalyst exhibited an 8.4-fold enhanced mass activity when compared to the Pd/C catalyst. In addition, the Pd metallene aerogel exhibited a higher power density in an ethanol single-cell fuel cell with a 19.1 mW cm^−2^ maximum power density, which is higher than the Pd/C catalyst. This indicates that Pd metallene can be a promising anodic electrocatalyst for DEFCs (Figure 9).

Mehdi et al. [76] investigated the effect of various solvents on the synthesis of Pd metallene aerogels by utilizing different types of carboxylic acid solvents such as alkyl groups (R=H– (formic acid, FA), CH_3_– (acetic acid, AA), CH_3_CH_2_– (propionic acid, PA), and CH_3_CH_2_CH_2_– (butyric acid, BA)). A comprehensive investigation revealed that the side chain of the alcohols significantly influences the thickness and morphology of the Pd nanosheets. It was found that, with a decrease in the length of the alkyl group, an enhanced self-assembly of the Pd nanoparticles was seen. It is believed that shortening the carbon chain will aid the self-assembly process because carboxylic acid solvents contain carbon chains that can function as capping agents. Since BA contains an alkyl group with three carbon atoms, which function as a gentle capping agent and inhibit the self-assembly process, the Pd hydrogel is only weakly formed when BA is used as the solvent. In contrast, Pd hydrogel is highly stable when dissolved in an FA solvent devoid of alkyl groups. Among the investigated solvents, the AA or PA solvents were found to acts as excellent capping agents, allowing the Pd nanoparticles to self-assemble in the form of nanosheets. Utilizing AA or PA as a solvent triggers a slower decomposition process, while carbon chains serve as a weak capping agent, enabling the Pd clusters to progress towards the creation of nanosheets. Similarly to the previously mentioned study [73], the authors employed CO gas as a reducing agent during the synthesis. CO gas bubbling and increased temperatures change the yellow color of the solution to dark green and then to black, suggesting the successful reduction of the Pd^2+^ ions into Pd atoms. The TEM measurements reveal ultrathin Pd nanosheets for Pd (AA) and Pd (PA) catalysts. The If/Ib ratios are 1.0 for Pd/C, 1.39 for Pd(BA), 1.72 for Pd(PA), 1.69 for Pd(AA), and 1.38 for Pd(FA). The higher oxidation currents suggest that Pd metallene synthesized with acetic acid exhibits superior electrocatalytic activity.

**Figure 10:**
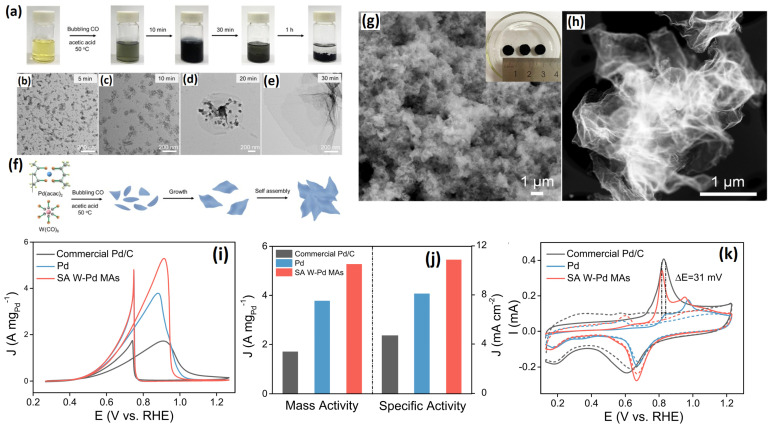
(**a**) Pictorial representation of SA W-Pd Mas; (**b**–**e**) TEM images of the SA W-Pd MA catalysts recorded at different time intervals; (**f**) schematic representation of the SA W-Pd MA catalyst synthesis; (**g**) SEM images of SA W-Pd MAs (inset: pictures of powder catalyst); (**h**) TEM images of SA W-Pd MA catalysts; (**i**) ethanol oxidation CV curves of various catalysts; (**j**) mass and specific activity values; (**k**) CO oxidation results of SA W-Pd MAs, Pd and commercial Pd/C catalysts. Reproduced with permission from Ref. [77].

Progressing towards the Pd metallene aerogel, Wang et al. [77] introduced the concept of single-atom doping to Pd metallene with tungsten (W). A CO gas-induced gelation strategy with acetic acid as the solvent and capping agent at 50 °C was used to synthesize W-doped Pd metallene aerogel catalysts (W–Pd) with a gelation time of 1 hr. After the addition of CO gas at 50 °C, a jelly-like black product was obtained, which upon supercritical drying generates the W-doped Pd catalysts. The SEM and TEM images show that the W-Pd catalyst shows highly porous 3D nanosheets with ultrathin sheets of 2.72 nm in thickness, which could offer excellent catalytically active sites due to its hierarchical nanosheet morphology. TEM elemental mapping clearly confirms the presence of W atoms inside the crystal lattice of the Pd, which was further confirmed by the AC-HAADF-STEM technique, with visible bright spots in the Pd metallene. The structural analysis indicates the potential integration of W species into the Pd lattice; however, the XPS results reveal that tungsten primarily exists in an oxidized W^6+^ state on the catalyst surface. Though the previous works suggest that CO gas is essential for obtaining Pd metallene structures, experimental proof was only established in this study. In addition to CO, it is crucial to identify the specific metal precursors required to achieve the desired metallene structures. When the metal precursors were varied from Pd(acac)_2_ to H_2_PdCl_4_, gelation was achieved, but the Pd metallene sheets were found to be irregular. The formation mechanism of Pd was ascertained by collecting the intermediate products at buffer time intervals and was analyzed using TEM measurements. It was first seen that, when CO gas bubbling was done, the bright yellow color changed to black, indicating the reduction of Pd atoms. At about 5 min, Pd-W nanosheets with irregular shape were seen, which were then gradually transferred into nanosheets. At about 30 min, well-defined nanosheets were obtained via self-assembly. During the CO gas bubbling, the Pd reacts with CO to form the Pd-CO complex, which upon decomposition forms Pd atoms at the initial stage. As the reaction proceeds, more and more Pd atoms absorb on pre-formed Pd atoms to form the Pd sheets. In addition to the role of CO as a reducing agent, it also acts as a structural directing agent by strongly adsorbing on the Pd (111) facet, allowing it to grow anthropically, during which the reduced W atoms incorporate into the Pd crystal lattice. In addition, continuous CO gas purging creates turbulence in the reaction mixture, helping in accelerating the Pd growth kinetics by enhancing the mass transfer of Pd^2+^ ions. The simultaneous occurrence of these step results in the formation of gel, which, upon supercritical drying, results in Pd metallene sheets [54,78]. After a detailed analysis of the Pd metallene formation mechanism, the W-doped Pd aerogel catalyst was subjected to an EOR activity assessment. In comparison to Pd (I_f_/I_b_ = 0.80) and commercial Pd/C (I_f_/I_b_ = 0.99), the I_f_/I_b_ ratio of the W-doped Pd metal aerogel catalyst (I_f_/I_b_ = 1.30) was found to be higher, suggesting that the CO oxidation process is effectively enhanced by the atomically dispersed W atoms. Surprisingly, the W-doped Pd aerogel catalysts exhibited higher ECSA and mass activities than the control catalyst Pd aerogels and commercial Pd/C catalyst. The incorporated W atoms helps in CO oxidation, assessed through CO stripping experiments where the CO electro-oxidation of the W-doped Pd catalyst was seen to occur at 31 mV lower than the control Pd aerogel catalysts, which demonstrates the enhanced anti-CO poisoning effect of W atoms (Figure 10).

#### 2.1.3. Bimetallic Pd Metal Aerogels

**Figure 11:**
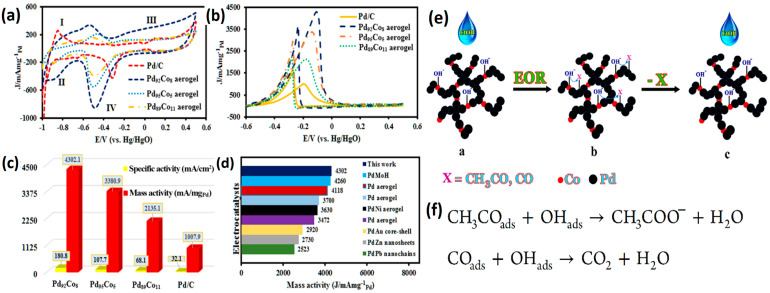
(**a**) CV curves and (**b**) ethanol oxidation reaction; (**c**) mass and specific activities curves of various Pd_x_Co_y_ catalysts; (**d**) comparison of mass activities of various Pd-based catalysts vs. Pd_92_Co_8_ catalyst; (**e**) schematic representation of oxophilic nature of Co and reaction mechanisms involved in anti-poisoning effect; (**f**) chemical reaction representing CO removal. Reproduced with permission from Ref. [82], open access.

The development of Pd bimetallic catalysts with enhanced EOR and stability is essential due to the high cost and scarcity of the noble metals. The alloying of Pd with other transition metals can be helpful in reducing the loading of noble metals and engineering the d-band center of the alloy. It is thought that alloying noble metals with other noble or transition metal elements will effectively control the electrical and geometric structure while simultaneously lowering the mass loading. Alloy catalysts provide significantly improved electrocatalytic activity, according to both computational and experimental studies [79,80,81]. Douk et al. [82] proposed a synthesis strategy of bimetallic Pd-Co aerogel via a controlled temperature-induced destabilization of the metallic nanoparticles. The metallic ions are reduced by using NaBH_4_ as a reducing agent. During the reduction of metallic nanoparticles, the generation of hydrogen is a pre-stage of the construction of aerogel. However, during the gelation process, isotropic strong electrostatic repulsion between precursor ions and pre-formed nanoparticles impedes the 3D gelation process. To overcome the isotropic repulsion, the temperature of the solution was raised to 343 K. Raising the temperature makes the surrounding environment more conducive to the disruption necessary for the NPs to be connected, which in turn produces the 3D hydrogels. With the temperature up, Brownian motion can be improved, and isotropic repulsions can be overcome, which speeds up the process of building 3D hydrogels. The metal aerogels obtained show an excellent 3D network generated by the expanded nanochains and excellent crystallinity. The variation in the Co atomic ratio with Pd shows that Pd_92_Co_8_ enhances ethanol oxidation currents, mass and specific activities. The 3D Pd_92_Co_8_ aerogel (107 m^2^/g) showed enhanced electrochemical activity compared to other Pd_x_Co_y_ compositions and commercial Pd/C catalysts. The ethanol electrocatalytic activity of the 3D Pd_92_Co_8_ aerogel catalyst showed the highest I_f_/I_b_ = 1.17 and enhanced onset potentials for ethanol oxidation. The observed enhanced ethanol electro-oxidation of Pd_92_Co_8_ is due to the interconnected pores and nanowire-based structure of the Pd_92_Co_8_ aerogel catalyst that guarantees the effective contact between the catalyst surface and EtOH reactants, which is crucial for heterogeneous catalysis. Furthermore, the role of Co as an oxophilic metal improves electrocatalytic performance and efficiently hinders CO poisoning. The EOR is known to generate reaction intermediates that poison the catalyst, such as CH_3_CO and CO. In order to remove these reaction intermediates, it is essential to have a secondary oxophilic element such as Co that provides the OH− species. The produced intermediates are eliminated more quickly due to the adsorption of OH− species. Once the CH_3_CO and CO intermediates are removed, the electrocatalyst’s active sites will be available for the uptake of additional ethanol molecules (Figure 11).

In another study, Yao et al. [83] proposed a unique Pd-CoO_x_ aerogel using an in situ electrochemical reduction strategy. Initially, the mixture of Pd and Co precursors was added to the Na_2_CO_3_ solution for 12 h to obtain the Pd-Co oxide hydrogel. The hydrogel was then subjected to supercritical drying to obtain the metal aerogel. Then, the Pd-CoO_x_ hydrogel was coated on the Ti plate, and the reduction of hydrogel was conducted using a chronoamperometric technique [84]. With the addition of an Na_2_CO_3_ solution for over 12h, the color of Pd-Co oxide gels gradually changed from brown to light blue. The reduction of the processor was monitored by using UV-Vis spectroscopy, which confirmed the reduction of the metallic species. The SEM and TEM images show the excellent interconnecting crystalline nanoparticles. In contrast to other noble metal aerogels, including pure Pd aerogels, the N_2_ physical adsorption isotherm of Pd-CoO_x_ showed a combination of type II and type IV curves, suggesting the presence of macropore structures with a specific surface area of 203.2 m^2^ g^−1^ [85,86,87,88]. Such a high surface area of the PdCoO_x_ aerogel could have resulted from the unique electrochemical reduction process. The TEM morphological analysis showed the well-maintained porous morphology, with crystal planes of Pd (111) and Co_3_O_4_ (311) planes recognized at the lattice d spacing values of 2.25 Å and 2.43 Å, respectively. Evidence for the electrochemical reduction of Pd and Co in the gel into the metallic phase was ascertained through the XPS analysis of the gel before and after the electrochemical reduction. Following the electrochemical reduction, the XPS analysis revealed that the peaks linked to metallic phases emerge, shifting the peaks linked with oxides. This suggests that the Pd element in Pd-Co oxide aerogel underwent a transformation from its ionic state into metallic Pd(0) [89]. However, the Co-oxide peaks did not fully transform into metallic Co, though a slight reduction in the Co^3+^/Co^2+^ was observed, indicating that the applied redox potential is not enough to reduce the Co species, and hence the Co is said to be present in the form of CoO_x_ rather than metallic cobalt. Pd_3_-CoO_x_ exhibited 7.4 times the mass activity of the Pd/C catalysts. The enhanced Pd_3_-CoO_x_ results from the unique 3D porous network and high specific surface area, which can promote the high metallic utilization. In addition, the metal/metal oxide interfacial junction enhances the electron transfer and induces a strong metal–support interaction (SMSI), and the oxophilic metals facilitate the -OH species for the desorption of reaction intermediates [90,91].

**Figure 12:**
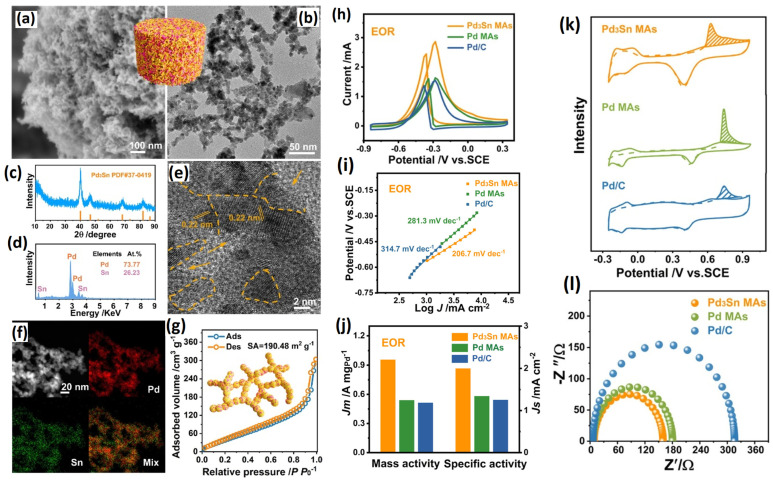
(**a**) SEM and (**b**) TEM images; (**c**) XRD patterns; (**d**) EDAX spectrum; (**e**,**f**) HR-TEM images and elemental mapping; (**g**) BET isotherm of Pd_3_Sn MAs; (**h**) CV ethanol oxidation reaction; (**i**) Tafel slopes; (**j**) mass and specific activities of Pd_3_Sn MAs; (**k**) CO oxidation CV curves and (**l**) impedance curves of Pd_3_Sn MAs, Pd MAs and Pd/C catalysts. Reproduced with permission from Ref. [98].

The presence of CO adsorbates on Pd surfaces during EOR is a drawback for electrocatalysts and can lead to CO poisoning. To alleviate CO poisoning, generally, an oxophilic element adjacent to the Pd is required. The orbital interaction between the oxophilic element and Pd atoms results in the removal of CO molecules from the Pd atoms. In particular, p-d orbital hybridization is found to be an effective strategy for the enhancement of the electrocatalytic property [92,93]. For example, Te-Pd, Sn-Ru, Ga-Ru and Sn-Ga catalysts were shown to enhance electrocatalytic activity via the C1 pathway [94,95,96]. Among these, Sn is an interesting, oxophilic element known for enhancing anti-poisoning ability and accelerating the oxidation of adsorbed CO and the activation of surface sites [97]. On the other hand, Sn has a lot of p orbitals. So, making Pd-Sn alloys can make Pd’s electronic structure controlled and lessen Pd poisoning by combining p and d orbitals in Sn. Most importantly, p-d orbital hybridization has the ability to control the strength of intermediate binding in electrochemical reactions, leading to an improvement in catalytic activity. In this regard, Liu et al. [98] proposed a Pd_3_Sn metal aerogel with p-d orbital engineering for boosting EOR. In a unique synthesis process, Pd metal aerogels were obtained by using ionic liquid as a gelation initiator and hydrazine hydrate as the reductant agent. Precursors of Pd and Sn can be advantageously co-reduced and gelated in a single step with the help of ionic liquid, which is essential for inhibiting the hydrolysis of metals [99]. The TEM measurements show the highly crystalline Pd_3_Sn phase with a lattice edge of 0.22 nm corresponding to the {111} facet of Pd_3_Sn phases. In particular, Pd_3_Sn metal aerogel was seen, and a high concentration of unsaturated sites and surface defects will increase the number of active sites and, to avoid agglomeration, will be beneficial in establishing numerous anchoring points to the methanol fuel on the surface of the electrode. The CO stripping experiments reveal an ECSA of 47.83 m^2^ g_Pd_^−1^, which is higher than those of Pd metal aerogel and commercial Pd/C (40.19 and 40.92 m^2^ g_Pd_^−1^). In an electrochemical analysis, the Pd_3_Sn metal aerogel showed the largest ethanol oxidation peak and mass and specific activities of 0.96 A mg_Pd_^−1^ and 2.01 mA cm_Pd_^−2^ for the EOR, confirming the positive effects of improving electrocatalytic performance through p-d orbital hybridization on the electronic structure of the catalyst Pd_3_Sn. In the anti-poisoning study via CO stripping experiments, the adsorbed CO oxidizes at the potential of 0.63 V vs. SCE for the Pd_3_Sn MAs and at a much lower potential than the Pd metal aerogels and Pd/C catalysts, suggesting that the Pd_3_Sn metal aerogels possess an excellent CO anti-poisoning ability that can help in more easily stripping off CO molecules for an enhanced EOR (Figure 12).

#### 2.1.4. Pt-Based Bimetallic Aerogels

**Figure 13:**
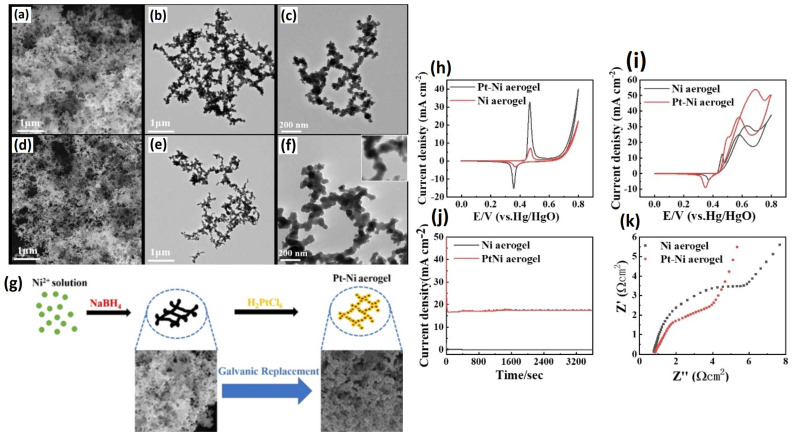
SEM and TEM images of Pt-Ni aerogel (**a**–**f**); (**g**) schematic of Pt-Ni aerogel synthesis; (**h**) CV of Pt-Ni aerogel in 1.0 M KOH; (**i**) CV in 1.0 M KOH and 0.1 M C_2_H_5_OH; (**j**) I vs. t curves; (**k**) Nyquist plots of different aerogels. Reproduced with permission from Ref. [100].

Pt-based metal aerogel catalysts are still regarded as efficient electrocatalysts for ethanol electro-oxidation. However, the high cost and low stability are major issues that affect the fast development of anode EOR electrocatalysts. Alloying the Pt with transition metals is generally considered a way to improve the electrocatalytic activity and reduce the Pt loading. Tang et al. [100] proposed Pt-Ni bimetallic metal aerogel catalysts. In this study, ultra-low Pt atoms are coated on bulk Ni metal aerogels, and this was found to enhance the electrocatalytic activity resulting from improved electronic conductivity. The PtNi aerogel was obtained using an in situ self-gelling strategy with NaBH_4_ as a reducing agent. TEM images show the 3D porous network structure where the nanowires of randomly arranged metal nanoparticles are connected to each other. When compared to Ni aerogel, the onset potentials and oxidation peak potentials for Pt-Ni aerogels are lower, suggesting that Pt significantly affects the electrical characteristics of the Ni aerogel. At 50.8 mAcm^−2^, the peak current density of the Pt Ni aerogel was approximately 1.35 times that of the Ni aerogel. Since Ni and Pt are clearly different in their electronegativity values, Ni gives Pt a 5d empty orbit when they are joined, while Pt gives Ni electron density, which lowers the Pt-Co bond energy. Consequently, the catalytic efficiency of Pt Ni aerogel for ethanol oxidation is enhanced because the adsorption of CO on the Pt surface is attenuated and the synergistic effect based on the bifunctional catalytic mechanism is accomplished. The enhanced EOR of Pt-Ni could be attributed to several factors, such as (i) the improved conductivity of the catalyst through ultra-low Pt coating on the Ni surface, (ii) the 3D porous network that allows the high exposure of the catalytic active sites and helps in improved electron transfer and mass transfer, and (iii) the oxophilic nature of Ni, helping in the desorption of reaction intermediates (Figure 13).

In another study, Zhang et al. [101] introduced Ru as an oxophilic element into a Pt lattice, aiming to improve the reduction in the adsorption of CO. By introducing Ru into the reaction, hydroxyl groups with a lower potential can be adsorbed on the active sites, where they are more effectively bound and where the activity and stability of the sites are enhanced, protecting them from CO poisoning. The Pt-Ru metal aerogels are prepared using the NaBH_4_ reduction method. The HR-TEM image shows that Pt_6_Ru MAs have abundant pores and defects, which can provide more active sites to improve their catalytic performance. Among different atomic proportions of metal aerogels, Pt_6_Ru metal aerogel can be seen, showing the best electrocatalytic activity with a mass activity higher than 1.8 times that of the Pt/C catalyst, in addition to the higher I_f_/I_b_, with a lowest Tafel slope of 162 mV dec^−1^. The H^1^ NMR spectra reveal that the only reaction product of ethanol oxidation is acetic acid. The XPS analysis revealed a substantial Pt peak shift in the PtRu metal aerogel catalysts compared with the PtRu nanoparticles, attributed to the strong electronic interaction between Pt and Ru [102]. In addition, the d-band center analysis revealed a positive shift for PtRu metal aerogel, and the shift is positively correlated with the content of Ru [103], suggesting that Ru plays an important role in the bifunctional mechanism of anti-CO poisoning caused by the weaker adsorption of CO species.

**Figure 14:**
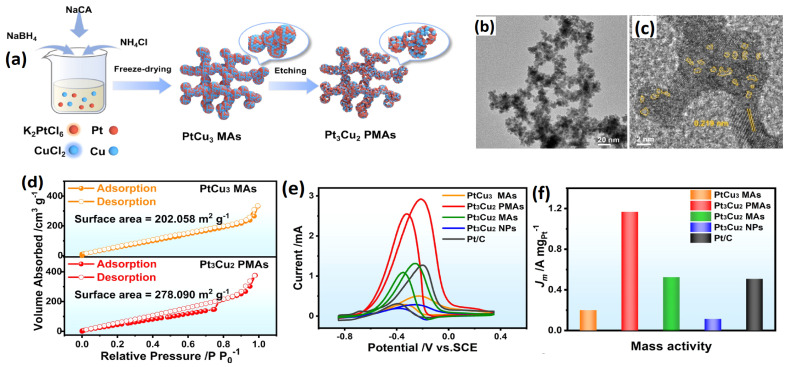
(**a**) Schematic representation of Pt_3_Cu_2_ MA catalyst synthesis; (**b**,**c**) TEM images of Pt_3_Cu_2_ MA catalyst; (**d**) BET isotherm; (**e**) methanol oxidation reaction CV curves; (**f**) mass activity values of various Pt-Cu metal aerogels. Reproduced with permission from Ref. [104].

One significant problem that limits the development of Pt-based catalysts is their susceptibility to surface poisoning by CO. The adsorbed CO species inhabit the surface-active sites, hindering the available electrochemical surface area for the consequent catalytic cycle. Zhang et al. [104] proposed an acid etching strategy to synthesize Pt_3_Cu_2_ porous metallic aerogel. First, the PtCu_3_ metal aerogel catalysts were synthesized, which were then subjected to acid etching, during which Cu oxidizes to CuO, and further etching at higher temperatures of 120 °C in acetic acid, resulting in the accelerated etching of Cu and in a porous Pt_3_Cu_2_ metal aerogel catalyst [105,106]. The acid-etched Pt_3_Cu_2_ metal aerogel catalysts show an excellent porous 3D network, crystallinity, revealed through XRD analysis, and clear evidence of acid etching on the Cu, leaving behind porous hollow space that can be noticed in the TEM measurements. The magnified HRTEM image shows lattice fringes with an interplanar spacing of 0.212 nm, corresponding to the (111) plane of Pt-Cu_3_ alloy, suggesting the high crystallinity of the catalysts. After treating pristine PtCu_3_ MAs with concentrated hydrochloric acid to remove Cu, the resulting Pt_3_Cu_2_ MAs retained their three-dimensional network structure. For the Pt_3_-Cu_2_ (111) plane, there are distinct lattice fringes of 0.219 nm. Notably, the Pt_3_Cu_2_ MAs’ XRD diffraction peaks have moved to lower angles, suggesting an expansion of the lattice structure (PtCu_3_ → Pt_3_Cu_2_). When compared to the d spacing of pure Pt (111) with 0.226 nm, the Pt_3_-Cu_2_ (111) d spacing of 0.219 confirms lattice contraction due to Cu incorporation into the Pt lattice. In addition, the acid etching resulted in an enhancement of the specific surface from 202 m^2^ g^−1^ to 278 m^2^ g^−1^. The enhanced surface area is due to the porous structure of the metal aerogels resulting from the Cu dissolution during the acid etching process. The mass activity of Pt_3_Cu_2_ PMAs is 1.17 A mgPt^−1^, which is 5.85, 2.23, 10.26, and 2.3 times higher than those of PtCu_3_ MAs (0.20 A mgPt^−1^), Pt_3_Cu_2_ MAs (0.52 A mgPt^−1^), Pt_3_Cu_2_ NPs (0.11 A mgPt^−1^), and commercial Pt/C (0.51 A mgPt^−1^), respectively (Figure 14).

While numerous metallic aerogels incorporating non-precious metals have been created for electrocatalysis, their application is primarily restricted to 3d transition metals like Cu, Co, and Ni. The optimization of electrocatalysis could be greatly enhanced by using 5d transition metals that exhibit higher spin coupling with noble metals. A 5d transition metal electrocatalyst is still in its early stages of development, nevertheless. Zhang et al. [107] introduced Hg into the Pt crystal lattice with controlled chemical composition via a facile one-step gelation method. An attractive three-dimensional structure made of interconnected extended nanochains with an average diameter of 9.0 nm characterizes the Pt-Hg electrocatalyst, which exhibits a high porosity. The Pt-Hg aerogel outperforms the commercial Pt/C by a factor of 10.5 due to its significantly higher current density and mass activity, reaching 9.05 A mgPt^−1^. Notably, the Pt-Hg aerogel exhibits a specific activity up to 12.47 mA cm^−2^, which is approximately 2.4, 2.7, 8.1, 2.7 and 7.5 times higher than that of Pt_3_Hg, PtHg_2_, PtHg_4_, monometallic Pt and Pt/C. The results show that adding the right quantity of mercury to Pt-based aerogels for electrocatalysis is crucial. The catalysts’ selectivity was further elucidated by assessing their electrocatalytic activity toward the oxidation of the EOR key intermediate, specifically CH_3_CHO. A lower onset potential for acetaldehyde oxidation on the PtHg aerogel (0.20 V) compared to the Pt aerogel (0.35 V) was noted, suggesting that the CH_3_CHO molecule oxidation can take place at a low potential on the Hg-doping catalyst. Consequently, the selective oxidation of ethanol to acetic acid can be explained by the fact that alloying with Hg is the main factor that enhances CH_3_CHO oxidation on Pt-Hg surfaces.

#### 2.1.5. Au-Based Bimetallic Aerogels

**Figure 15:**
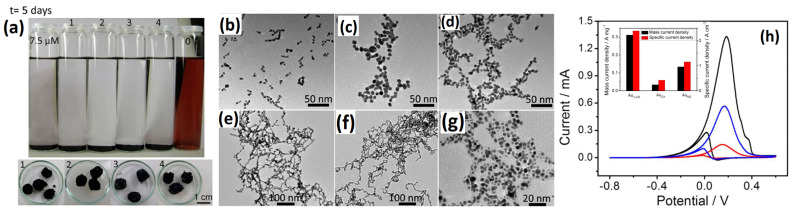
(**a**) Pictures of Au aerogel gelation over time and its corresponding powders; (**b**–**g**) TEM images of β-CD Au aerogel; (**h**) ethanol oxidation cyclic voltametric curves (inset: mass activity values of β-CD Au aerogel (black), Au aerogel (blue) and Pd/C (red). Reproduced with permission from Ref. [108], open access.

In contrast to other noble metal aerogels such as Pd and Pt mono- and bimetallic metal aerogels, the reports on aerogels with Au specifically are few due to the fact that Au metal aerogel synthesis involves a longer gelation time and the ability to aggregate into larger nanoparticles of a size more than 100 nm [45]. In order to mitigate Au nanoparticle aggregation during the synthesis process, Wen et al. [108] described a dopamine-mediated self-assembly of Au into 3D nanowire-like networks with a particle size as low as 5–6 nm. In this unique synthesis process, Au aerogel was synthesized by using β-CD, citrate and non-stabilizers (without organic surface ligands) as capping agents. During the reduction of Au*^n+^* precursors, β-CD absorbs onto the growing nanoparticles and, due to its large macromolecular structure and negative charge, stabilizes the nanoparticles [109]. In addition, according to reports, dopamine has the ability to create a host–guest inclusion complex with -CD in aqueous solution. Additionally, due to the high affinity between dopamine and the Au core, it may also bind and interact with the surface of Au. A hydrogel might be formed by inducing the β-CD–Au NPs to combine using dopamine as a destabilizing agent. After the addition of the dopamine, the gelation was started and a fluffy black solid containing the Au hydrogel was obtained after 6h, and it was also found that the gelation time can be modified by changing the concentration of dopamine. The produced Au aerogel reveals a web of bifurcated, extremely porous, and linked structures resembling very thin wires. With both meso- and macropores open, their pore size distribution is quite wide. The Au aerogels show a polycrystalline, face-centered, cubic crystal arrangement assessed via XRD analysis. It was observed that the host–guest interaction between β-CD and dopamine is one of the driving forces for Au hydrogel formation. By accumulating TEM images at various gelation times, the source of the Au metal aerogel network was determined. It was observed that dopamine induces the fusion of nanowires within 2 h, starting with small assemblies of several NPs and progressing to short nanowires, small, branched networks, and finally largescale interconnected nanowire networks. There was no evidence of aggregation after 2 days, and densely linked Au nanowires of about 5–6 nm were visible. When the β-CD was replaced by other stabilizers such as pyrocatechol, 2-phenylethylamine hydrochloride, ferrocene carboxylic acid, and citrate, a different morphology of the Au morphologies was obtained. When the Au aerogels with different morphological structures were analyzed for ethanol oxidation reactions, the Au aerogel obtained from the β-CD was shown to have higher ethanol oxidation current, mass and specific activities compared to Au NP morphologies, suggesting that the host–guest chemistry between β-CD and dopamine is an essential factor in obtaining Au metal aerogels with a smaller particles size, resulting in a higher ECSA and consequently higher electrocatalytic activity (Figure 15).

In a landmark work, Du et al. [110] proposed an excessive reductant-directed gelation strategy to synthesize all the eight noble metals in a combined theoretical and experimental work. In addition, the effect of counter-anions and reductants, including initiations, stabilizers and reducing agents, has been investigated in detail. The NaBH_4_ concentration for the metal precursor ratio (R/M) between 1.5 and 5.0 is well reported [111,112,113,114]. However, in this study, an R/M of 100 has been adopted. In contrast to gel formation of about a few weeks to 6 h at 333 K [111,112], in this study, the gel is formed at room temperature within 4–6 h, which is achieved by using excessive NaBH_4_. By manipulating the R/M ratio, the stability of colloidal solutions of metal NPs are investigated. It was found that, at a high or low R/M ratio (>50 (or) <2), the destabilization of the NP solution was observed, whereas, with a medium R/M ratio, a stable Au solution was observed. Based on these observations, NaBH_4_ functions differently at different concentrations.

At low concentrations (R/M < 2), the NaBH_4_ functions only as a reducing agent. During the reduction, the Au NPs form and are fused together to form agglomerated, larger Au NPs.

At medium concentrations (R/M < 50), after the excess NaBH_4_ reduces all the metallic precursors, the excess dissociated/decomposed products of NaBH_4_ into BH_4_^−^ or BO_2_^−^ essentially act as a stabilizer, in addition to being the reductant.

At higher concentrations (R/M > 50), the NaBH_4_ has a salting-out function, in addition to being the reductant and stabilizer. At higher concentrations, the NaBH_4_ acts as an electrolyte by initiating nanoparticle aggregation and fusion in the presence of in situ-formed decomposed products of NaBH_4_ into BH_4_^−^ or BO_2_^−^. The salting-out function of an excessive NaBH_4_ naturally acts as a destabilizing agent to quicken the gelation of the NPs into hydrogel.

**Figure 16:**
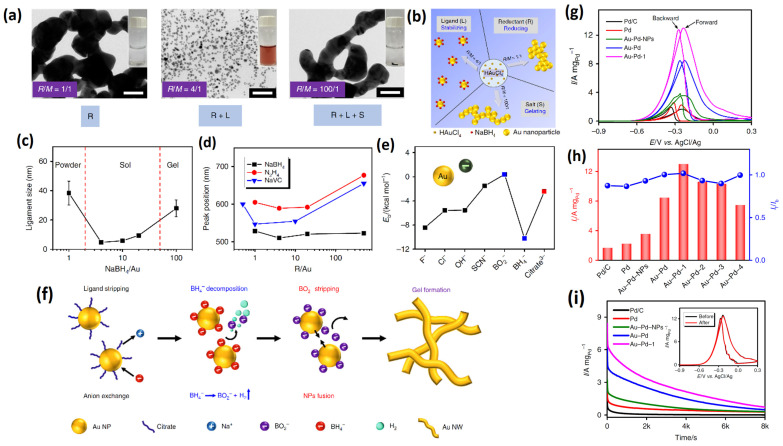
(**a**) TEM images of Au aerogel at different R/M ratios; (**b**) role of sodium borohydride; (**c**) relationship between ligament size vs. NaBH_4_/Au; (**d**) UV-vis max for various NaBH_4_/Au; (**e**) binding energy for various anions; (**f**) mechanism of gelation of Au; (**g**) EOR performance; (**h**) mass activity vs. I_f_/I_b_ for various Au-Pd systems; (**i**) i vs. v reposes for various Au-Pd catalyst systems. Reproduced with permission from Ref. [110], open access.

The salting-out effect of excessive NaBH_4_ was discovered and cross-verified using other not-dissociating reductants such as hydrazine, sodium hypophosphite and sodium ascorbate. Even at double the concentration of these reductants (vs. NaBH_4_) at R/M > 200, they only act as reducing agents, not as destabilizing agents. Hence, this work clearly established the potential use of excessive NaBH_4_ reductant as a reducing agent and gelation initiator. The salting-out effect was due to the anions that are generated from the decomposition of NaBH_4_ (BO_2_^−^). When the reductants with different anions were investigated, such as F^−^, Cl^−^, OH^−^, SCN^−^ and citrate anions, only the BO_2_^−^ showed excellent gelation kinetics. This synthesis process was then extended to various noble metal aerogel systems such as Pd, Ru, Rh, Os and noble metal alloys such as Au-Pd, Au-Ag, Au-Ru, and Au-Ir aerogels. The as-obtained selected metal aerogels have been applied as electrocatalysts for ethanol oxidation. Among several catalysts, Au-Pd exhibited the highest ethanol oxidation currents and a higher mass activity compared to other catalyst, including commercial Pd/C catalysts (Figure 16).

Aerogels with multiple metals have the potential to increase composition/structure variety, leading to the elaboration of new functions or the stimulation of desirable synergistic effects, in comparison to MAs with a single metal component [56]. Most metal aerogel syntheses proceed through the addition of reductants to the concentrated solutions, which results in uncontrolled metallic structures. It is still not clear how to regulate the size variation in ligaments and nanochains, which determines a number of nano-effects that have a significant impact on how well they work in applications. In particular, when metal aerogels are subjected to catalyst ink preparation under ultrasonication conditions, there is a high probably that the long metallic network might undergo disintegration. In a recent study, Liu et al. [115] proposed an integrated well-preserved structure of Pt-Ag aerogel on a macropore polymer framework via a silicon limited gelation approach that delivered a high electrocatalytic activity for the methanol oxidation reaction. The disadvantage of this method could be the high possibility of contamination from silicon. On the other hand, it is well reported that the gelation process and gelating agents significantly affect the morphology of metal aerogels [110]. In a recent study, Cui et al. [116] investigated a template-free, multimetallic effect-directed approach to engineer the sol–gel process at room temperature. The controlling sol–gel process and ligament size (d_L_) were manipulated by altering the average bulk density of the alloy and mismatch of the atomic radius of Au and M (M = Pt. Ru and Cu). Based on the high density of Au (19.32 g cm^−3^), the authors utilized the natural gravitation-based sedimentation of the gel formation. After the formation of nanoparticles (by excess NaBH_4_ reduction), gelation was achieved through the sedimentation process, during which the formed metal aggregates gradually settled down and enriched at the bottom of the vessel. The higher the bulk density of the metal, the faster the sedimentation process and gel formation. UV-vis spectroscopic studies reveal that most of the large aggregates form within 15 min, with the large metallic aggregates forming faster than small aggregates. In addition to the effect of gravity on sedimentation leading to gel formation, a unique ligament length modification strategy was unveiled by adding a smaller quantity of secondary metals. It was found that, with an addition of 1% of the secondary metal salts, the ligament d_L_ was reduced by 30–78% (from 43.9 to 9.6 nm). The d_L_ was further reduced by increasing the proportion of the secondary metal, leading to a d_L_ as low as 6 nm. It is worth noting that a size-focusing effect was observed with the introduction of several kinds of metal independent of their exact composition; this provides a simple approach to precisely tailoring d_L_. The varied d_L_ affected the specific surface area and in turn the electrocatalytic activity. With a bimetallic aerogel of Au-M, the dL was 9.6–30.6 nm, whereas, for multimetallic aerogel, it was reduced to −13 nm. Therefore, just by adding the metallic salts of different metallic species, it is easier to obtain a customized d_L_ for MMAs by adding metallic salts rather than manipulating the d_L_ by using organic ligands [117]. The reduction in the d_L_ was correlated to the atomic radius (r_s_) mismatch between Au and the secondary metal. When the lattice increases, the mismatch d_L_ of the corresponding Au-M aerogels monotonously decreases. The growth mechanism was explained based on either Frank–van der Merwe (FM) or Volmer–Weber (VM)-like nanomaterial growth. The FM-grown model involves the addition of atoms layer-by-layer on the existing atom of the same r_s_, whereas the VM model involves the addition of atoms on the existing atom of a different r_s_ with a distorted crystal lattice [118]. In general, the addition of an atom to the metal surface requires sufficient chemical potential [119]. In a single Au system, the thickening of the ligament is highly possible due to energetically favored atomic growth with the similar metal surface (FM growth). However, the different precursor metals require a higher chemical potential, leading to the shift in the growth mechanism from the FM to the VM model, leading to the formation of branched ligaments, thus leading to the smaller d_L_. The viability of multimetallic effects controlling both the sedimentation rate and d_L_ has been demonstrated in widespread MMA systems, since similar results were seen when this hypothesis was expanded to multimetallic systems. The non-destructive method, employed by keeping the carbon paper at the bottom of the vessel and allowing the sediment to settle on the CP, forming CP-protected intact gel film, can be used further for electrocatalytic activity evaluation. The model catalyst Au_50_Pt_50_ gel was subjected to methanol and ethanol oxidation reactions and showed an excellent oxidation current, with a 17-times-higher mass activity than the Au_50_Pt_50_ and Pt/C catalyst.

**Figure 17:**
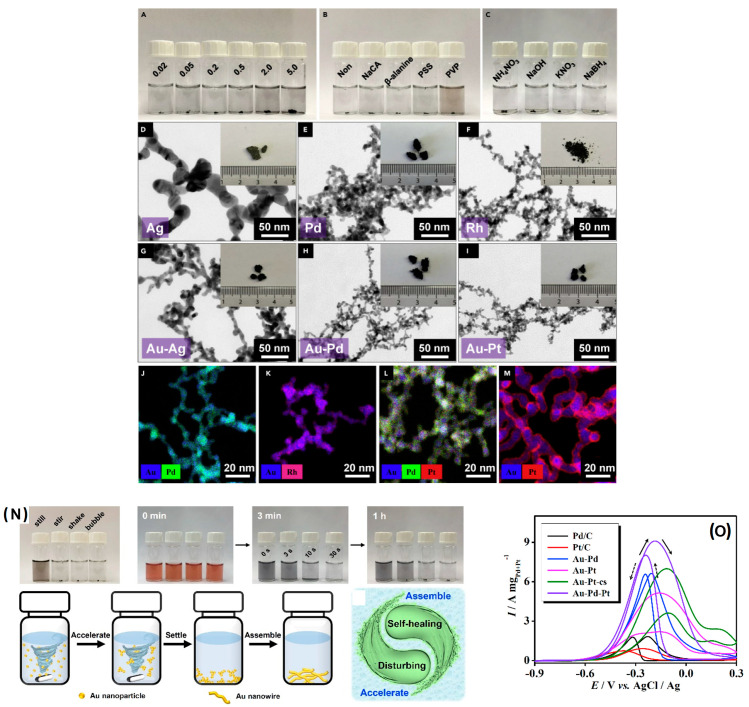
(**A**–**C**) General overview of disturbance-promoted gelation; (**D**–**M**) TEM images of various metal aerogels; (**N**) photographs of various Au catalysts under different treatment conditions over time. Proposed model for stirring-promoted gelation and the function that the self-healing characteristics and the disturbed environment played in this process. (**O**) EOR activity of various metal aerogels. Reproduced with permission from Ref. [120], open access.

Despite the tremendous development in metal aerogel catalysts since their discovery in 2009, one factor still remains a big challenge: controlling the gelatin time, especially with low to medium precursor ion concentrations (especially C_M_ < 1.0 mM) at room temperature. In this regard, recently, Du et al. [120] proposed a unique self-healing, force field (disturbance)-induced gelation of Au metal nanoparticles to accelerate gelation time to as low as 1–10 min. Applying the force field via disturbance of the metallic solution by stirring was found to shorten the gelation time, even at lower concentrations (C_M_ = 0.02–5.0 mM). The general gelation process includes the assembly of nanoparticles at static conditions, which can be highly limited by mass transfer of the nanoparticles from the bulk to the self-assembly site due to the large diffusion barrier, which significantly slows down the overall gelation process. The slight disturbance to the solution containing nanoparticles breaks the diffusion limit during the gelation process even at very low ionic concentrations. This concept was initially proposed for organic gel systems [121,122]. However, for the first time in a study, it was shown that, even for inorganic systems, the concept can be swiftly applied. With NH_4_F as an initiator and salting-out agent, it was seen that large aggregates were formed within 45 s and a clear monolith gel was formed within 4 min, assisted by stirring. Disturbance has the potential to dramatically improve mass transfer, which in turn lowers the diffusion barrier and speeds up the gelation process. The proposed disturbance-induced gelation process was then translated from a simple Au metal aerogel system to other metal aerogel systems such as Ag, Pd, Rh, Au-Ag, Au-Pd, Au-Pt and Au-Pd-Pt; it was found that the concept worked well with other metal aerogel systems, indicating the universality and generality of the applied concept (Figure 17). The synthesized catalysts were applied as anode catalysts for the ethanol oxidation reaction, and the trimetallic system of Au-Pd-Pt exhibited the highest oxidation currents.

#### 2.1.6. Ag-Based Bimetallic Aerogels

Metal aerogel systems with Pt and Pd are popular and proven to be excellent electrocatalysts, with enhanced durability due to the compositional and morphological effects of their electronic structure, alternation in the d-band center, and the effects of the secondary metal acting as an oxyphilic element [123,124]. By combining the electrocatalytic activity of silver (Ag), an oxyphilic metal, with platinum (Pt) and palladium (Pd), an alkaline environment can be used for the successful electro-oxidation of alcohol. The enhanced electrocatalytic capabilities of the Pd-Ag and Pt-Ag electrocatalysts in binary form are credited to the alteration in electronic structure brought about by their combined actions [125,126]. Douk et al. [127] synthesized a Ag-Pt/Pd catalyst through a surfactant-free and reductant-free approach utilizing the galvanic displacement reaction (GDR) of Ag NPs (as template) and M^2+^ (M^2+^ = Pt^2+^ and Pd^2+^) ions, followed by the initiation of anisotropic environments, to form the 3D gels. The Ag-M metal aerogel was synthesized by reducing metallic salts via electrochemical reduction. A smart approach was used in which sacrificial Ag NPs were generated by taking a naturally decomposing silver-format salt. The Ag salt naturally decomposed at temperatures less than 5 °C. To the Ag-containing solution, a solution of Pt^2+^ and Pd^2+^ ions were added, which resulted in a spontaneous reduction of the Pt^2+^ and Pd^2+^ into metallic species due to electron transfer from Ag to Pt^2+^ and Pd^2+^. Ag’s lower reduction potential than Pt/Pt^2+^ and Pd/Pd^2+^ resulted in the generation of colloidal Pd-Ag nanoparticles, which were then destabilized by elevating the temperature. The increase in temperature induced the Brownian motion of the NPs, helped to overcome the isotropic repulsion, and enhanced the diffusion rate of the NPs from the bulk to the site of nanoparticle fusion. The resulting Pd-Ag aerogel and Pt-Ag aerogels show excellent crystallinity, alloying behavior, and 3D porous interconnected nanoparticles. During the electrocatalytic activity evaluation of Pd-Ag aerogel and Pt-Ag aerogels, these catalysts showed strong ethanol oxidation peaks and I_f_/J_b_ values of 0.6, 2.67, and 1.38 for Pd/C, Pt-Ag aerogel, and Pd-Ag aerogel, respectively. Both aerogels seem to be more poison-tolerant than Pd/C according to these results.

In the synthesis of Ag-based aerogels, the self-assembly of Ag nanoparticles generally results in a ligament size (d_L_) of 18–50 nm, which closely depends on the NP assembly and fusion kinetics dictated by the initiating methods [128,129,130]. For the Ag aerogel from the assembly of Ag NWs by freeze-casting, the d_L_ was found to be as high as 30–250 nm when compared to the other noble metal aerogels such as Au, Pt and Pd [57,61]. The intriguing nano-effects of Ag aerogels are stifled by their huge d_L_, which is likely caused by the lower atomic diffusion barrier of Ag owing to its low cohesive energy [131]. Went et al. [132] proposed a unique strategy to synthesize customized Ag aerogels with a d_L_ range of 8.4–134.4 nm by regulating metal–ion, metal–ligand, and metal–metal interactions. The hypothesis was based on controlling d_L_ via tuning the metallic ion-containing solution by taking advantage of anisotropic electrostatic repletion and isotropic van der Walls attraction from the ion–metal interaction [54] by introducing specific ion-containing initiators. It was found that the d_L_ of the Ag aerogels seems to strongly depend on the type of anions used, such as F^−^, OH^−^, and Cl^−^. This could be explained by the significant destabilizing ability of salting-out anions, which leads to a high reaction activity of Ag NPs and, as a result, causes them to adapt to a diffusion-limited cluster-building model. The rapid coupling of NPs results in the formation of a gel network that is highly branched, which in turn results in the formation of tiny ligaments [133]. It is well documented that surface growth of the nanoparticles can be tuned through metal–metal interactions by leveraging the chemical potential of different metals [119]. Based on this concept, the secondary metal Ni was used to tune the d_L_ of Ag metal aerogels to essentially shift the growth of Ag aerogels from FM to VM growth, leading to the formation of branched ligaments with a smaller d_L_. As expected, the Ag aerogels’ d_L_ significantly reduced from 71 to 8.4 nm with the addition of Ni from 0.5 to 50 at.%. This clearly suggests that the secondary metal helps to enhance the branched growth of Ag metal aerogels. In general, the axial and radical orientational growth of metal aerogels regulate the size/shape of the nanomaterials, with particular facet and growth kinetics [134]. A tiny ligament and a large aspect ratio are predicted outcomes of axial growth that significantly outpaces radial growth. In this aspect, considerable interaction between metal and ligand is paramount to guarantee interparticle crosslinking, which is fundamental for obtaining a gel. In this work, the authors explored a variety of ligands, including cationic, anionic and non-ionic ligands, to investigate the effect of the surface interaction of metal on the Ag metal aerogel d_L_ size. Among these, cationic ligands gave the largest d_L_ size, anionic ligands gave a medium d_L_ size and non-ionic ligands gave the lowest d_L_ size for Ag metal aerogels, suggesting the role of the secondary metal and low-concentration ligands in obtaining Ag metal aerogels with smaller and highly branched d_L_ sizes. In addition, the proposed concept is further ratified by its universality in synthesizing a variety of Ag-M aerogels (M = Au, Pd, Pr, Ir, Rh, PdAu, PtPdAu). This work reveals the extraordinary energy-related application potential of wide metal aerogels and provides instructions for modifying their multiscale architectures.

**Figure 18:**
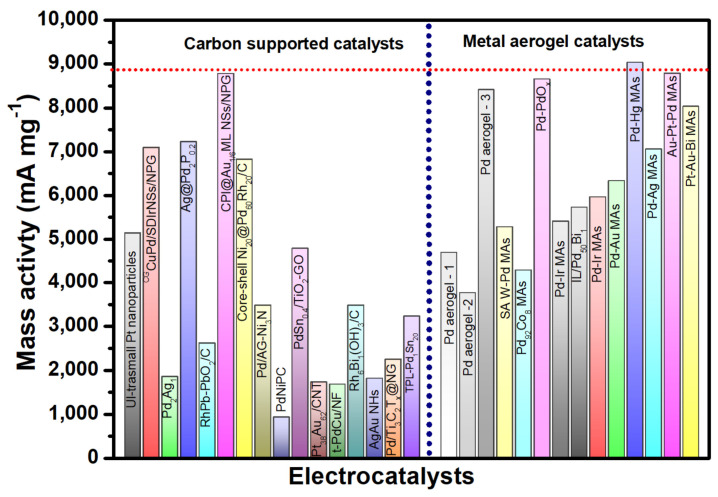
EOR performance of carbon-supported vs. metal aerogel catalysts. The catalyst names and corresponding references are as follows. Ultrasmall Pt nanoparticles [141], ^CG^CuPd/SDIrNSs/NPG [142], Pd_2_Ag_1_ [143], Ag@Pd_2_P_0.2_ [144], RhPb-PbO_2_/C [145], CPI@Au_1/6_ML NSs/NPG [146], core–shell Ni_20_@Pd_60_Rh_20_/C [147], Pd/AG-Ni_3_N [148], PdNiPC [149], PdSn_0.4_/TiO_2_-GO [150], Pt_38_Au_62_/CNT [151], t-PdCu/NF [152], Rh_6_Bi_1_(OH)_3_/C [153], AgAu NHs [154], Pd/Ti_3_C_2_T_x_@NG [155], TPL-Pd_1_Sn_20_ [156], Pd aerogel-1 [88], Pd aerogel-2 [66], Pd aerogel-3 [73], SA W-Pd MAs [77], Pd_92_Co_8_ MAs [82], Pd-PdOx [83], Pd-Ir MAs [135], IL/Pd_50_Bi_1_ [87], Pd-Ir MAs [85], Pd-Au MAs [136], Pd-Hg MAs [107], Pd-Ag MAs [127], Au-Pt-Pd MAs [138] and Pt-Au-Bi MAs [140].

Table 1 details metal aerogel synthesis and EOR electrochemical kinetic data in comparison to the standard Pd/C and Pt/C catalysts [135,136,137,138,139,140] and clearly shows that metal aerogels outperform in EORs, suggesting their potential as efficient catalysts for EORs. From the data given in Table 1, it can be seen that most of the studies have been performed in alkaline electrolytes, and it is noticeable that noble metal aerogels outperform the traditional Pd/C catalysts both in terms of I_f_/I_b_ and mass activities. In addition, the data shown in Table 1 can be used to compare the potential of metal aerogels with traditional carbon-supported catalysts [141,142,143,144,145,146,147,148,149,150,151,152,153,154,155,156], as shown in Table 2 and Figure 18. It is seen that metal aerogel catalysts show challenging competition in terms of mass activity with traditional carbon-supported catalysts. In addition, metal aerogel catalysts contain no carbon in addition to having a simple synthesis process, and hence metal aerogel catalysts still represent one of the potential candidates in relation to traditional carbon-based catalysts. With no carbon as support, it is believed that metal aerogels alleviate the stability issues generally associated with carbon-supported catalysts.

**Figure 19:**
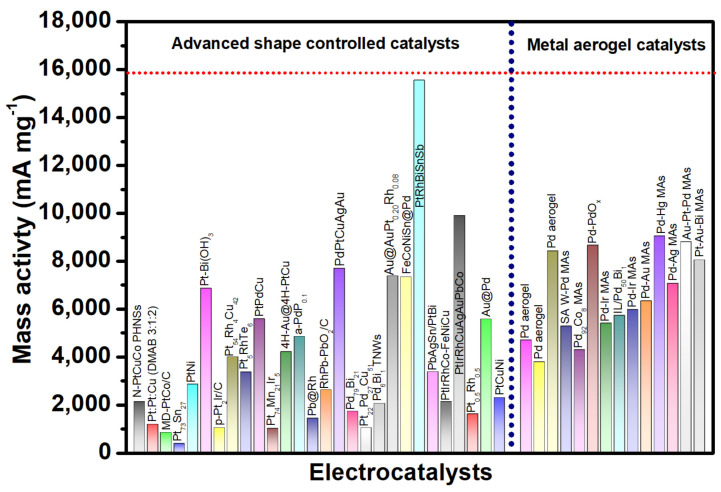
EOR performance of advanced shape-controlled catalysts vs. metal aerogel catalysts. The catalyst names and corresponding references are as follows. N-PtCuCo PHNSs [157], Pt:Pt:Cu (DMAB 3:1:2) [158], MD-PtCo/C [159], Pt_73_Sn_27_ [160], PtNi [161], Pt-Bi(OH)_3_ [162], p-Pt_2_Ir/C [163], Pt_54_Rh_4_Cu_42_ [164], Pt_5_RhTe_6_ [165], PtPdCu [166], Pt_74_Mn21Ir_5_ [167], 4H-Au@_4_H-PtCu [168], a-PdP_0.1_ [169], Pb@Rh [170], RhPb-PbO_2_/C [171], PdPtCuAgAu [172], Pd_79_Bi_21_ [173], Pt_22_Pd_27_Cu_51_ [174], Pd_6_Bi_1_ TNWs [175], Au@AuPt_0.2_Rh_0.08_ [176], FeCoNiSn@Pd [177], PtRhBiSnSb [178], PbAgSn/PtBi [179], PtIrRhCo-FeNiCu [180], PtIrRhCuAgAuPbCo [181], Pt_0.5_Rh_0.5_ [182], Au@Pd [183], PtCuNi [184], Pd aerogel-1 [88], Pd aerogel [66], Pd aerogel [73], SA W-Pd MAs [77], Pd_92_Co_8_ MAs [82], Pd-PdO_x_ [83], Pd-Ir MAs [135], IL/Pd_50_Bi_1_ [87], Pd-Ir MAs [85], Pd-Au MAs [136], Pd-Hg MAs [107], Pd-Ag MAs [127], Au-Pt-Pd MAs [138], and Pt-Au-Bi MAs [140].

Furthermore, the potential of metal aerogel catalysts was also compared with other advanced electrocatalyst systems such as state-of-the-art nanowires, hollow nanostructures, multicubes, core–shell systems, nanoframes, 1D nanotubes, nanodendrites and high-entropy catalysts [157,158,159,160,161,162,163,164,165,166,167,168,169,170,171,172,173,174,175,176,177,178,179,180,181,182,183,184], as shown in Table 3 and Figure 19. It can be seen that metal aerogel catalysts’ mass activities are well aligned with the state-of-the art advanced catalysts. It is observed that some of the advanced, shape-controlled catalysts show relatively higher mass activities compared to metal aerogels. However, the shape-controlled catalysts require a very high precision in the synthesis process, often requiring carefully chosen rection conditions, surfactant capping agents, etc., and are generally considered very challenging to scale-up suitably for industrial applications [185].

Based on this, metal aerogel catalysts come with the special advantages of an easier synthesis process and scalability. Therefore, metal aerogel-based electrocatalysts have potential as efficient electrocatalysts for ethanol electro-oxidation compared to shape-controlled electrocatalysts.

### 2.2. Factors Responsible for Enhanced EOR Activity of Metal Aerogels

**Figure 20:**
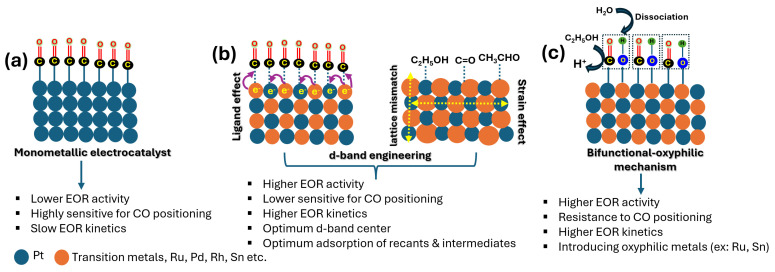
(**a**) CO poisoning on Pt surface; (**b**) d-band engineering of alloy catalysts; (**c**) bifunctional-oxophilic mechanism to remove adsorbed CO species.

The enhanced EOR of the metal aerogels primarily arises from the nanochain type of self-supporting morphology, with a continuous metallic skeleton that results in enhanced electron transport across the 3D hierarchical structure. In addition to the morphological advantage, the enhanced mass and specific activities of the metal aerogel electrocatalysts also originate from the intrinsic catalytic performance of a given catalytic surface and are fundamentally determined by the electronic structure and coordination environment of the surficial active sites. Some of the important factors that are responsible for enhanced EOR activity are (i) tuning the d-band center and (ii) bifunctional oxophilic effect mechanisms, as described below (Figure 20).

(i) Tuning the d-band center: The d-band center of the electrocatalysts determines the energy of the d-band center relative to the fermi levels, which determines the strength between the reaction intermediates and the surface of the electrocatalysts [186,187]. The strong adsorption of reaction intermediates slows down the catalytic cycle, whereas the weaker adsorption leads to insufficient reaction kinetics, thus requiring that the catalysts should have an optimum energy that is just right, meaning not too strong and not too weak. The d-band center helps identify the optimum energy of the reaction intermediates. The d-band center optimization is performed using alloying (ligand effect) and the lattice strain (geometric effects) [188,189].

The ligand effect is ascertained either by introducing secondary metals into the nanoparticles of the metal to form alloys or by modifying the surface of noble metals with surface capping agents (ex. polymers or small organic molecules), which is considered a widely accepted phenomenon. The d-band theory generally validates that the downshift of the d-band center is usually associated with the weakened (towards the optimum side) interaction between the reaction intermediates such that -CO decreases, benefiting the overall activity enhancement [190]. On the other hand, if the d-band center is downshifted too far from the fermi level, it drastically weakens the interaction between the metal active sites, and the adsorbates result in a decreased reaction efficiency. Therefore, the coordination environment of the metallic sites, the composition, and the electronic interaction between the alloying elements significantly affect the position of the d-band center. For several metal aerogel catalysts that we discussed in the above sections and that are based on bimetallic, trimetallic, and multimetallic alloys, the ligand effect is primarily responsible for the enhanced EOR activity. Furthermore, the compressive strain induced by the alloying elements with different lattice parameters leads to a mismatch in the curvature stress in the junction of the nanoparticles, lowering the d-band center, while the tensile strain usually lifts the d-band center closer to the fermi level, therefore enhancing the interaction with the reaction intermediates. The tensile strain also greatly facilitates surface -OH formation to help remove CO. The strain effect is an important design criterion for EOR electrocatalyst [191].

(ii) Bifunctional mechanism and oxophilicity: In the EOR, CO poisoning is one of the critical challenges. CO removal is primarily accomplished through the L-H mechanism, wherein CO on noble metal sites reacts with adjacent -OH groups rather than with free OH- anions [192,193]. Nevertheless, noble metals exhibit low oxophilicity and therefore cannot efficiently promote the adsorption of -OH groups for optimal CO elimination. Consequently, a crucial design criterion is the incorporation of oxophilic components (e.g., Ni(OH)_2_, Ni, Ru, Sn, Ti, etc.) to facilitate CO removal. The introduction of oxophilic elements to noble metals was proven to be a straightforward solution to alleviate CO oxidation. The oxophilic element catalyzes water activation to provide -OH_ads_ to assist in the removal of -CO from the strongly adsorbed active sites.

In conclusion, the enhanced EOR activity of metal aerogels arises from their unique nanochain type of self-supporting morphology, with a continuous metallic skeleton that results in enhanced electron transport across the 3D hierarchical structure and electronically modifying the catalyst surface for optimum ethanol adsorption and oxidation, together with a bifunctional mechanism to remove the adsorbed CO with the help of oxophilic elements.

**Table 1:** Metal aerogel synthesis and EOR electrochemical kinetic data.

Metal Aerogel Catalyst	Reducing Agent/Gelation Time	BET/ECSA Surface Area (m^2^ g^−1^)	Electrolyte	I_f_/I_b_ of Metal Aerogel Catalyst	I_f_/I_b_ vs. Standard Catalyst	Mass Activity of Aerogel Catalyst	Mass Activity vs. Standard Catalyst	Ref.
Pdα-CD aerogel	NaBH_4_	92/69	1.0 M KOH + 1.0 M C_2_H_5_OH	1.28	1.09 (Pd/C)	NR	NR	[48]
Pd	Na_2_CO_3_ and C_2_H_2_O_3_. H_2_O	50/133	1.0 M KOH + 0.5 M C_2_H_5_OH	1.59	0.6 (Pd/C)	4.703 A mg^−1^	NR	[88]
Pd aerogel	C_2_H_5_OH/NaOH 2 h	44/75	1.0 M KOH + 1.0 M C_2_H_5_OH	1.53	0.6 (Pd/C)	3787 mA mg^−1^	1074 mA mg^−1^	[66]
Pd aerogel	CO gas/2 days	75/77	1.0 M KOH + 0.5 M C_2_H_5_OH	1.48	0.6 (Pd/C)	8425.2 mA mg^−1^	1007.9 mA mg^−1^	[73]
Pd (FA) formic acid	CO gas/4 h at 50 °C	NR	1.0 M KOH + 1.0 M C_2_H_5_OH	1.38	1.0 (Pd/C)	284 mAcm^−2^ (oxidation current)	78.1 (oxidation current)	[76]
Pd (PA) propionic acid	CO gas/4 h at 50 °C	NR	1.0 M KOH + 1.0 M C_2_H_5_OH	1.72	1.0 (Pd/C)	478 (oxidation current)	78.1 (oxidation current)	[76]
Pd (BA) (butyric acid)	CO gas/4 h at 50 °C	NR	1.0 M KOH + 1.0 M C_2_H_5_OH	1.39	(Pd/C)	380 (oxidation current)	78.1 (oxidation current)	[76]
Pd (AA) (acetic acid)	CO gas/4 h at 50 °C	NR	1.0 M KOH + 1.0 M C_2_H_5_OH	1.69	1.0 (Pd/C)	533 (oxidation current)	78.1 (oxidation current)	[76]
SA W-Pd MAs	CO gas/1 h at 50 °C	44/48	1.0 M KOH + 1.0 M C_2_H_5_OH	1.30	0.99 (Pd/C)	5.29 A mg_Pd_^−1^	3.1 times lower (Pd/C)	[77]
Pd_92_Co_8_ aerogel	NaBH4/1.83 h at 70 °C	32/107	1.0 M KOH + 0.5 M C_2_H_5_OH	1.17	0.6 (Pd/C)	4302 mA mg^−1^	1008 mA mg^−1^	[82]
Pd-CoO_x_	Na_2_CO_3_/12 h	203/52	1.0 M KOH + 1.0 M C_2_H_5_OH	NR	NR	8.67 A mg^−1^	7.4 times lower	[83]
Pd3Sn MAs	N_2_H_4_·H_2_O/rest until hydrogel forms	190/47	1.0 M KOH + 0.5 M C_2_H_5_OH	NR	NR	0.96 A mg^−1^	NR	[98]
Pd-Ir aerogel	NaBH_4_/2.41 h, 60 °C	42/68	1.0 M KOH + 0.5 M C_2_H_5_OH	1.45	0.6 (Pd/C)	5416 mA mg^−1^	1007 mA mg^−1^	[135]
IL/Pd_50_Bi_1_ hydrogels	NaBH4/1 h at 60 °C	46/59	1.0 M KOH + 1.0 M C_2_H_5_OH	1.03	0.73 (Pd/C)	5.74 A mg^−1^	1.40 mA mg^−1^	[87]
Pd-Ir aerogel	HCHO, Na_2_CO_3_/~2 h at 60 °C	57/90	1.0 M KOH + 0.5 M C_2_H_5_OH	1.49	0.6 (Pd/C)	5977.7 mA mg^−1^	1008 mA mg^−1^	[85]
Pd-Au aerogel	C_2_H_2_O_3_, Na_2_CO_3_/5 h at 60 °C	90/136	1.0 M KOH + 0.5 M C_2_H_5_OH	1.86	0.6 (Pd/C)	6344 mA mg^−1^	1007 mA mg^−1^	[136]
Pt6-Ru MAs	NaBH_4_/12–24 h	99/13	1.0 M KOH + 0.5 M C_2_H_5_OH	Higher I_f_/I_b_ of Pt_6_-Ru MAs than Pt/C		1.264 A mg_Pt_^−1^	0.696 A mg_Pt_^−1^	[100]
Pt_3_Cu_2_ PMAs	Sodium citrate, NH_4_Cl, NaBH_4_	278/54	1.0 M KOH + 0.5 M C_2_H_5_OH	Higher I_f_/I_b_ of Pt_3_Cu_2_ PMA MAs than Pt/C		1.17 A mg _Pt_^−1^	0.51A mg _Pt_^−1^	[101]
PtHg aerogel	NaBH_4_/8 h at 60 °C	27/23	1.0 M KOH + 1.0 M C_2_H_5_OH	Higher I_f_/I_b_ of PtHg aerogel MAs than Pt/C		9.05 A mg _Pt_^−1^, 10.5-times-higher mass activity		[107]
Auβ-CD	Dopamine, β-CD 6 h	50/18	1.0 M NaOH + 1.0 M C_2_H_5_OH	5.1	NR	0.31 A mg^−1^	NR	[108]
Au-Pd	NaBH_4_/4–6 h	~60/NR	1.0 M NaOH + 1.0 M C_2_H_5_OH	1.5 times higher than Pd/C		3.79 A mg^−1^	1.68 A mg^−1^	[110]
Au_50_Pt_50_ gel	NaBH_4_/12 h	NR	1.0 M NaOH + 1.0 M C_2_H_5_OH	Higher I_f_/I_b_ than the Pt/C		~2.8 times improvement		[116]
Au-Pd-Pt	NH_4_F/Na_3_C_6_H_5_O_7_ 90 s/2–3 days	42.6/NR	1.0 M NaOH + 1.0 M C_2_H_5_OH	1.4	NR	1.8 A mg_Pd_^−1^ 8.8 A mg_Pd+Pt_^−1^	0.9 A mg_Pt_^−1^	[120]
Pd-Ag aerogel	Galvanic displacement reaction (GDR)	NR/83	M NaOH + 0.5 M C_2_H_5_OH	Higher If/Ib of Pd-Ag aerogel than Pt/C		7066.7 mA mg_Pd_^−1^	1007.9 mAmg _Pd_^−1^	[127]
Au/Ag/Pd aerogel	NaBH_4_/16–24 h	269/	1.0 M NaOH + 1.0 M C_2_H_5_OH	8 times higher I_f_/I_b_		∼20−30 times increase in current density		[137]
Au-Pt-Pd gel	NaBH_4_/1 h	6.5/83	1.0 M NaOH + 1.0 M C_2_H_5_OH	1.15	1.19	8.8 A mg^−1^_Pt+Pd_	1.14 A mg^−1^_Pt+Pd_	[138]
Ag/Pt/Pd-2	NaBH_4_/2–3 days of aging	119/NR	1.0 M NaOH + 1.0 M C_2_H_5_OH	Higher oxidation currents for MAs over Pd/C		5-times-higher mass activity for aerogel (2444.5 mA/mg) over Pd/C		[139]
Pt-Au-Bi aerogel	NaBH_4_/8 h	29/115	1.0 M NaOH + 1.0 M C_2_H_5_OH	Higher oxidation currents for MAs over Pd/C		8045 mA mg_Pt_^−1^	1064 mA mg_Pt_^−1^	[140]

NR = not reported.

**Table 2:** EOR activity of carbon-supported electrocatalysts.

Electrocatalyst	Mass Activity (mA mg^−1^) for EOR	Reference
Ultra-small Pt nanoparticles	5150 mA mg^−1^	[141]
^CG^Cu_1_Pd_1_/SDIr_0.03_ NSs/NPG	7105 mA mg^−1^	[142]
Pd_2_Ag_1_ nanosheets	1866 mA mg^−1^	[143]
Ag@Pd_2_P_0.2_	7240 mA mg^−1^	[144]
RhPb-PbO_2_/C	2636 mA mg^−1^	[145]
CPI@Au_1/6ML_ NSs/NPG	8796 mA mg^−1^	[146]
Core–shell Ni_20_@Pd_60_Rh_20_/C	6835 mA mg^−1^	[147]
Pd/AG-Ni_3_N	3500 mA mg^−1^	[148]
PdNiP/C	949 mA mg^−1^	[149]
PdSn_0.4_/TiO_2_-GO	4800 mA mg^−1^	[150]
Pt_38_Au_62_/CNTs	1746 mA mg^−1^	[151]
t-PdCu/NF	1695 mA mg^−1^	[152]
Rh_9_Bi_1_(OH)_3_/C	3500 mA mg^−1^	[153]
AgAu nanohybrids	1834 mA mg^−1^	[154]
Pd/Ti_3_C_2_T*_x_*@NG	2262 mA mg^−1^	[155]
TPL-Pd_1_Sn_20_	3246 mA mg^−1^	[156]

**Table 3:** EOR activity of shape-controlled advanced electrocatalysts.

Electrocatalyst	Morphology	Mass Activity (mA mg^−1^) for EOR	Reference
N–PtCuCo PHNSs	Nanowire	2140 mA mg_Pt_^−1^	[157]
Pt^2−^:Pt^2+^:Cu (DMAB,3:1:2)	Macrobeams and macrotubes	1200 mA/g^Pt^	[158]
MD-PtCo/C	Nanoporous	850 mA/mg_Pt_	[159]
Pt_73_Sn_27_	3D nanostructure	≈400 mA mg_Pt_^−1^	[160]
PtNi	Multicubes	2860 mA/mg_Pt_	[161]
Pt-Bi(OH)_3_	Nanoframes	6870 mA/mg_P_	[162]
p-Pt_2_Ir/C	Nanocrystals	1019 mA/mg_pt_	[163]
Pt_54_Rh_4_Cu_42_	Cubic nanoboxes	4000 mA/g_Pt_	[164]
Pt_5_RhTe_6_	1D nanotubes	3370 mA/mg_pt+Rh_	[165]
PtPdCu NDs	Nanodendrites	5590 mA/mg_PtPd_	[166]
Pt_74_Mn_21_Ir_5_	Nanowires	1020 mA mg^−1^	[167]
4H-Au@4H-PtCu	Nanoribbons	4220 mA mg^−1^	[168]
a-PdP_0.1_	Amorphous palladium-based alloy nanoparticles	4851 mA mg^−1^ metal	[169]
Pb@Rh	Core–shell	1454 mA mg^−1^	[170]
RhPb–PbO_2_/C	Carbon-supported nanoparticles	2636 mA mg^−1^	[171]
PdPtCuAgAu	High-entropy nanowire	7700 mA mg^−1^_Pd+Pt_	[172]
Pd_79_Bi_21_	Nanochains	1740 mA mg^−1^_Pd_	[173]
Pt_22_Pd_27_Cu_51_	Ultrathin nanowires	1050 mA mg^−1^_PtPd_	[174]
Pd_6_Bi_1_ TNWs	Ultrathin twisty nanowires	2066 mA mg^−1^_Pd_	[175]
Au@AuPt_0.20_Rh_0.08_	Core–shell nanowires	7380 mAmg^−1^_Pt_	[176]
FeCoNiSn@Pd	High entropy alloy	7340 mA mg^−1^_Pd_	[177]
PtRhBiSnSb	Hexagonal close-packed High-entropy intermetallic	15,558 mA mg^−1^_Pt+Rh_	[178]
PdAgSn/PtBi	High-entropy alloy NPs	3386 mA mg_(Pd+Pt)_^−1^	[179]
PtIrRhCoFeNiCu	High-entropy alloy NP nanodendrites	2130 mA mg _Pt_^−1^	[180]
PdPtCuAgAuPbCo	High-entropy alloy nanowires	9900 mA mg_Pd+Pt_^−1^	[181]
Pt_0.5_Rh_0.5_	Hollow-structured	1629 mA g_PtRh_^−1^	[182]
Au@Pd	nanoframe@arrays	5580 mA mg^−1^,	[183]
PtCuNi	Nanohexapods	2310 mA mg_Pt_^−1^	[184]

## 3. Conclusions

Metal aerogels represent advanced nanomaterials composed entirely of 3D metallic networks. The general synthesis of metal aerogels involves a traditional sol–gel synthesis process, in which a solution containing ionic metallic precursors is reduced to metallic states using reducing agents. The resulting colloidal solution containing the metallic species is then destabilized using various destabilizing agents, allowing the self-assembly of metallic nanoparticles into hydrogels. Subsequent supercritical drying or freeze-drying of the hydrogel produces metallic aerogels with excellent interconnectivity and porosity. The metal aerogels do not contain any carbon support, resulting in a higher durability. A variety of metal aerogels, mostly from the noble group metals, have been proposed as excellent electrocatalytic materials for ethanol oxidation with a higher oxidation current and stability. Most importantly, metal aerogels exhibit excellent mass activity, typically 5–30 times higher than that of traditional Pt/C and Pd/C catalysts. This suggests that their highly exposed metallic active sites and three-dimensional porous structures result in excellent active site utilization. Several metal aerogel catalyst systems have also shown excellent anti-poisoning effects against CO species. In addition, metal aerogels exhibit the excellent porosity and surface area required for heterogeneous electrocatalysis. Overall, metal aerogel catalysts show excellent activity for the EOR and have the potential to replace traditional Pd/C- and Pt/C-based catalysts in commercial applications.

## 4. Challenges and Future Perspectives

**Figure 21:**
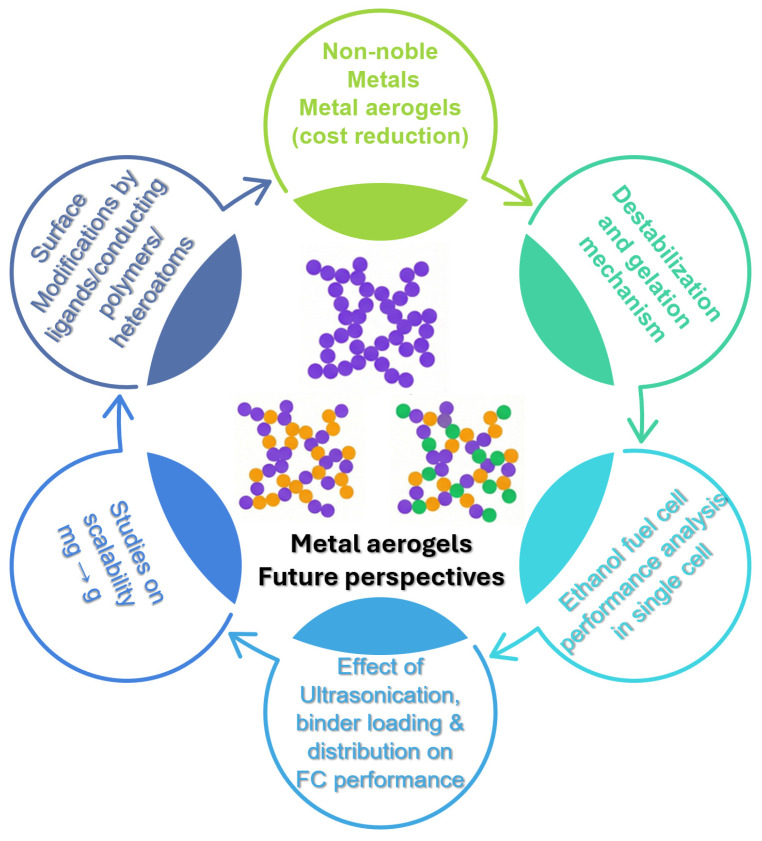
Pictorial representation of metal aerogel catalyst future perspectives.

A summary of future research perspectives for metal aerogel catalysts is given in Figure 21.

1. It is well established that the type of reductant used during metal aerogels synthesis influences the morphology, size distribution, and self-destabilizing behavior of the resulting materials. To date, most metal aerogels have been prepared entirely or largely using NaBH4 as a reductant. Therefore, it is essential to identify alternative reductants that might change the morphology and size distribution of the metal aerogel catalysts.
2. One of the important steps of metal aerogel synthesis is the destabilization of colloidal nanoparticles to obtain gels. Over the past decade, the gelation time for metal aerogels has been greatly reduced, from a few weeks to a few hours, due to continuous research efforts. However, knowledge on the mechanism of gelation has largely remained unknown, except for a few examples, such as NaBH_4_. Therefore, it is essential to understand the destabilization and gelation mechanism with a combined theoretical and experimental approach in order to tune the gelation step and control the morphology, size and growth of metal aerogels.
3. Despite significant advancements and the number of high-performance metal aerogels, there are still two significant obstacles to overcome. First, a significant obstacle to simple material production is the lack of a sufficiently quick gelation process (e.g., <10 min) for a low-to-medium metal concentration (e.g., cM < 1.0 mM) at ambient temperature. Therefore, efficient gelation processes need to be developed.
4. Since their discovery in 2009, all research until now has only been concentrated on noble metals and their combinations and few transition metals. Although noble metal aerogels exhibit excellent mass activities, these catalysts still contain noble metals and therefore remain expensive for commercial applications. Thus, it is essential to explore non-noble metal aerogels that might truly reduce the catalyst cost.
5. Ethanol oxidation is known to procced via a 12-electron transfer for complete oxidation. Hence, it is essential to understand whether or not metal aerogels proceed through a 12-electron transfer. To date, few studies have clearly reported the number of electrons transferred during the EOR over metal aerogels. Therefore, detailed studies are required for analyzing the potential of metal aerogels in comparison with traditional catalysts for complete or incomplete oxidation reactions.
6. The commercial potential of metal aerogel catalysts will only be recognized when they are applied as anode catalysts in realistic direct ethanol fuel cells, for which recording power density curves is paramount. Unfortunately, none of the studies have been dedicated to this direction. We believe that half-cell characterization of the catalyst in a traditional three-electrode system gives a fundamental idea of the activity of the catalysts; however, realistic applications will only be realized through a fuel cell power density analysis.
7. When analyzing the potential of metal aerogels, it is essential to coat the catalyst layer onto the carbon substrate of the gas diffusion layer. This requires the preparation of catalyst ink through ultrasonication. However, it remains unclear how the three-dimensional network morphology of metal aerogels changes during sonication. It is highly likely that the metallic network disintegrates, losing the advantages of metal aerogel physicochemical properties. Therefore, dedicated studies are required to investigate the morphological changes that occur during ink preparation. Recommendations about ultrasonication power, time and ink preparation protocols are also needed.
8. Furthermore, during the ink preparation process, the addition of a binder and its effect on the blockage of the metallic active sites remain unclear. The effects of ionomer loading and distribution in metal aerogels may differ from those in traditional carbon-supported catalysts.
9. Although metal aerogels exhibit a high mass activity, they are prone to undergoing coalescence, resulting in a reduction in catalyst stability. So far, most studies have focused primarily on the synthesis of metal aerogels, while only limited attention has been given to stability testing. Therefore, it is highly essential to comprehend their stability behavior and recommend standard operating protocols. After examining several research works on metal aerogel catalysts for the EOR, we found that almost all the research works predominantly used chronoamperometry (I vs. t) as a tool to measure the stability of the catalysts. Unfortunately, the stability tests were carried out for relatively short time periods. Furthermore, the stability tests were only done in a traditional three-electrode system in liquid electrolytes. We believe that current studies do not reflect the stability of metal aerogel catalysts in realistic experimental conditions such as fuel cell conditions and potential cycling conditions.
10. It is highly recommended to develop novel in situ metal aerogel MEA coating processes that mitigate the disadvantages of the effects of ultrasonication on the structural and morphological changes to metal aerogel catalysts.
11. To date, the yield of metal aerogels has generally been limited to milligram levels. In order to realize the commercialization status of metal aerogels, it is important to scale up metal aerogel synthesis from mg to gram levels.
12. Further improvements in metal aerogel catalysts could be achieved through surface medication techniques, either by ligands/conducting polymers, which may help in the stabilization of metal aerogels against coalescence. In addition, the modification of metal aerogels with heteroatoms such as N, S, P, B, and F could substantially improve ethanol oxidation kinetics and catalyst stability.
13. One of the important challenges in applying metal aerogels in MEAs is their impact on catalyst porosity and catalyst layer thickness. When MEAs are made using a hot-press, there is a high change that the intrinsic porosity of the metal aerogels may be compressed, which could affect the mass transfer of reactants and products. In addition, due to no carbon, the metal aerogels will significantly reduce the catalyst layer thickness. Therefore, systematic studies on catalyst layer thickness and catalyst loading optimization are highly required.

In conclusion, metal aerogel catalysts demonstrate potential applications in ethanol-based energy conversion devices such as direct ethanol fuel cells. They are attractive due to their good electrochemical activity toward ethanol oxidation, high surface area, scalability, hierarchical porous frameworks, and tunable characteristics.

## Data Availability

No new data were created or analyzed in this study. Data sharing is not applicable to this article.
